# Efficient mechanochemical synthesis of new fluorinated Schiff bases: a solvent-free, alternative to conventional method with mercury adsorption properties

**DOI:** 10.1186/s13065-025-01552-9

**Published:** 2025-07-04

**Authors:** Mirza T. Baig, Mariam T. Sayed, Reem Aledamat, Sumyah Hassan, Alaa AlReyashi, Naheed Sidiq, Siham Y. Al-Qaradawi, Mohamed F. Mady

**Affiliations:** Department of Chemistry and Earth Sciences, College of Arts and Sciences, Qatar University, Doha, Qatar

**Keywords:** Green chemistry, Schiff bases, Ball milling, Fluorine organo-compounds, Solvent-free reaction, Mercury adsorption

## Abstract

The development of sustainable synthetic techniques is of critical importance in modern chemistry. This paper describes a mechanochemical approach to the synthesis of novel fluorinated Schiff bases via ball milling, which provides a fast, high-yield, and green alternative to conventional reflux methods. The synthesized fluorinated Schiff bases are evaluated for their adsorption capacity and efficiency in mercury removal and compared to assess their performance, demonstrating that ball milling produced compounds with comparable physical attributes to those produced via traditional solvent-based procedures. However, ball milling considerably shortened reaction time, with some reactions taking less than 5 min, and enhanced yields, reaching up to 92%, which is significantly higher than that achieved by conventional methods. The molecular structure of the synthesised Schiff bases is validated using analytical and spectroscopic techniques, including ^1^H, ^13^C NMR and mass spectroscopy, with chemical shifts confirming the expected structures. Furthermore, the thermal stability of the synthesized Schiff bases is validated up to around 250 °C, demonstrating their robustness and suitability for various applications. Given their well-established chelating properties, the selected Schiff bases (**M6-M9**) are studied for mercury adsorption. Structural variations, such as hydroxyl and carbonyl functionalities, are anticipated to enhance mercury binding, making these compounds potential candidates for environmental remediation. Notably, this research presents the first investigation of these specific fluorinated Schiff bases for mercury chelation, demonstrating notable adsorption for mercury and highlighting their potential in heavy metal remediation.

**Graphical Abstract:**

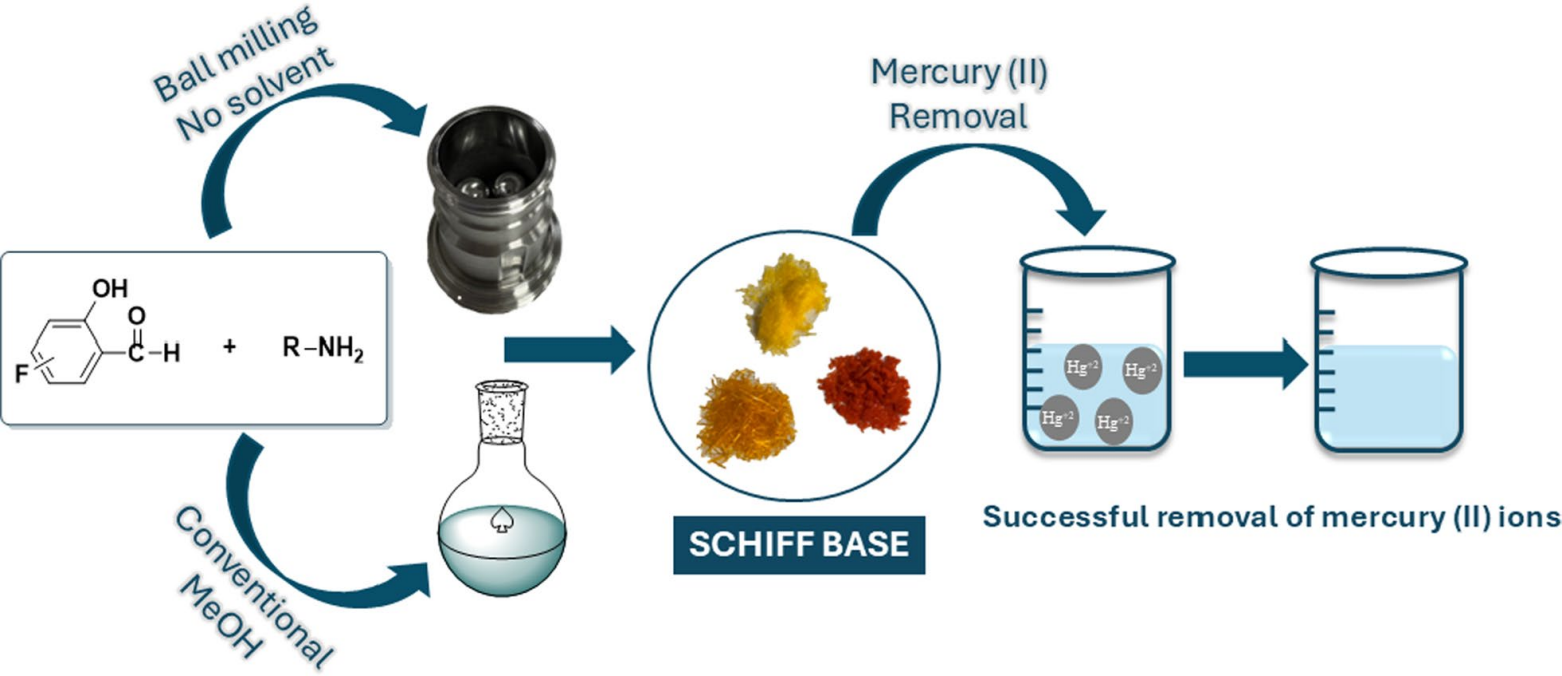

**Supplementary Information:**

The online version contains supplementary material available at 10.1186/s13065-025-01552-9.

## Introduction

The widespread contamination of water is largely caused by the rapid advancement of industrial technology. Various strategies have been used to reduce these harmful pollutants, aiming to protect ecosystems and human health. Heavy metal ions have become a pressing environmental issue due to their durability and accumulation, which pose a serious risk to both the environment and living organisms [1–3]. These toxic heavy metals have emerged from electronics, food, cosmetics, plastics, pesticides, and more [4]. Exposure to these pollutants can lead to severe health issues, including skin disorders, liver damage, and other chronic conditions, underscoring the urgent need for effective water treatment solutions [5]. The heavy metal mercury (Hg), which is frequently discovered in the crustal rocks and coal deposits of the Earth, is considered to be one of the most dangerous heavy metals due to its persistence in the environment and its ability to bioaccumulate [6]. Mercury exists in four main forms: elemental mercury (Hg^0^), inorganic mercury (Hg^2+^), methylmercury (MeHg), and various organic mercury compounds [7]. Industrial activities, including mining, fossil fuel combustion, and chemical manufacturing, are major sources of mercury pollution, leading to contamination of water and soil [8, 9]. Serious health problems, including brain impairment, kidney malfunction, and others, can be caused by exposure to mercury. Given its long-lasting environmental impact, developing effective and sustainable mercury removal methods is crucial to protect human health and the environment [10].

Various methods are employed to remove heavy metals from industrial wastewater, including ion exchange [11], membrane processes [12], adsorption [13], electrochemical techniques [14], and chemical precipitation [15]. Among these, adsorption stands out as one of the most widely used techniques due to its simplicity, cost-effectiveness, and ease of recovery, making it a highly efficient approach for metal ion removal. Various adsorbents are used, such as activated carbon, zeolites, metal–organic frameworks, and natural materials like clay and biosorbents. Each type of these adsorbents has its unique advantages, such as high surface area, adjustable pore sizes, or eco-friendliness. Additionally, silica gel and nanoparticle-based adsorbents have gained significant attention due to their high efficiency, large surface area, and the ability to be tailored for specific applications, making them promising candidates for the removal of metal ions, including mercury [16, 17]. Schiff bases have recently gained attention as an effective adsorbent due to their strong chelating properties, which enable them to form stable complexes with heavy metal ions [18].

**Fig. 1:**
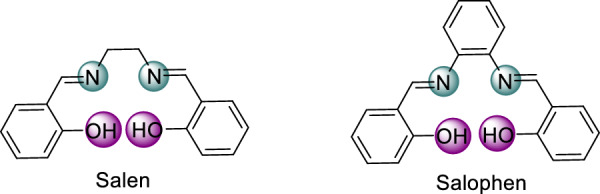
Chemical structures of Salen and Salophen ligands

Schiff bases are significant in coordination chemistry due to their ability to form stable complexes with metal ions. They are a class of compounds formed by the condensation of an aldehyde or ketone with a primary amine [19]. They are valued for their unique coordination properties and for their simple synthesis, versatility, and ability to stabilize metals in various oxidation states [20–22]. Their metal complexes are highly valued due to their stability, tunability, and wide range of applications [23–25]. Traditionally synthesized in solvents like methanol, these methods pose environmental and health risks due to solvent toxicity and pollution [26, 27]. Schiff bases find applications in pharmacology, optical materials, catalysis, corrosion inhibition, and metal chelation. [28–30]. Their applications in metal ion adsorption and removal, particularly for toxic metal ions like mercury, have been widely explored [31–33]. Salen and Salophen are classic examples of Schiff base ligands (Fig. 1).

To address these environmental concerns, there has been a growing emphasis on the adoption of green chemistry principles. Green chemistry emphasizes redesigning chemical processes to minimize hazardous substances, promoting safer, sustainable practices [34]. Mechanochemical synthesis, particularly ball milling, has gained attention as a solvent-free alternative. This method eliminates harmful solvents while providing faster reactions, improved yields, and high product purity, aligning with green chemistry principles [35].

Fluorinated compounds, while commonly used in medicinal chemistry, [36–38] also have significant environmental relevance [39]. The introduction of fluorine atoms into organic molecules enhances their stability and resistance to degradation, making them effective in adsorption processes for removing toxic metal ions like mercury [40].

This study investigates **5-fluoro-2-hydroxybenzaldehyde** and **4-fluoro-2-hydroxybenzaldehyde** as precursors for novel fluorine-based Schiff base compounds. These fluorinated benzaldehydes are increasingly recognized as versatile intermediates in pharmaceutical synthesis and environmental applications [41]. 5-Fluoro-2-hydroxybenzaldehyde has recently been employed to construct benzimidazole derivatives with potent antimicrobial and anticancer activities. Its role in biocatalytic processes enables the synthesis of fluorinated intermediates for antiviral and anti-inflammatory therapeutics. Beyond drug development, this compound has been utilized in positron emission tomography (PET) imaging agents for cancer diagnostics and in antimicrobial coordination complexes. Notably, it also serves as a ligand in Schiff base frameworks for copper ion detection and environmental remediation [42–46]. Similarly**,** 4-fluoro-2-hydroxybenzaldehyde has been leveraged to synthesize metal-based complexes with antioxidant properties, suggesting therapeutic potential for oxidative stress-related disorders [47]. These studies underscore the dual utility of fluorinated benzaldehydes in advancing both pharmaceutical innovation and environmental sustainability.

In this study, we explore the green, solvent-free synthesis of new and known various fluorinated Schiff bases using 4-fluoro-2-hydroxybenzaldehyde and 5-fluoro-2-hydroxybenzaldehyde with various primary amines via ball milling. This mechanochemical method aligns with sustainability principles, offering reduced reaction times, improved yields, and the elimination of harmful solvents. The resulting Schiff bases, known for their strong metal chelation properties, will be evaluated for the first time as mercury sorbents in aqueous solutions, emphasizing their potential for water treatment applications [48]. This work highlights the environmental benefits of green synthesis and the critical role of Schiff bases in heavy metal remediation.

## Experimental

### Materials

All chemicals used were purchased from VWR, Sigma-Aldrich or Merck and were used without purification. Ultrapure water, produced from an ultrapure water device (Stak Pure), was used in all experiments. Ball milling reactions were carried out in a Retsch CryoMill in a 25 ml stainless steel cell, and 3 stainless steel balls (12 mm) were used. Ball milling was performed at 30 Hz frequency at room temperature without the circulation of liquid. All reactions were monitored by thin-layer chromatography using Kieselgel 60 F254 sheets with detection under UV light at 254 and 360 nm. FTIR was measured using a Spectrum 400 FTIR from PerkinElmer using UATR with a range of 400 to 4000 and with a resolution of 4 cm^−1^. NMR spectra were recorded on a Joel NMR spectrometer at 600 and 151 MHz, at ambient temperature unless otherwise stated. ^1^H NMR and ^13^C NMR spectra were recorded in deuterated chloroform (CDCl_3_) or dimethyl sulphoxide (DMSO-d_6_) using TMS as internal standard. Shimadzu UV-2600i UV–Vis spectrometer, operated with LabSolutions UV–Vis software.

### Synthesis of Schiff bases

#### Conventional method

In a typical conventional reaction, a solution of the aldehyde 10 mmol, 1 eq in methanol was added to an equimolar amount of amine in methanol and placed under stirring at room temperature for the respective time. The reaction mixture was left until the reaction was complete, and the reaction was monitored via TLC. The TLC analysis was performed using a solvent system of [Hexane/Ethyl acetate (3:1)], which effectively separated the target compound from other components. The final product obtained was filtered and washed with cold EtOH and recrystallized from EtOH.

#### Ball milling method

In the ball milling method, 1.0 mmol of aldehyde was added to an equimolar amount of the amino compound in a mortar and a colour change was observed. The mixture was then ground together at room temperature for the respective time needed 5–30 min. The progress of the reaction was monitored by TLC every 5–10 min. After completion, the product was collected by filtration, then washed with cold EtOH and recrystallized with EtOH.

##### 4-Fluoro-2-(((3-hydroxyphenyl)imino)methyl)phenol (M1)

Yellowish orange; mp: 143–145 ℃; Yield: 83%; **IR υ**_**max**_ (cm^−1^): 3315 (OH), 1625 (C = N); ^**1**^**H NMR** (600 MHz, DMSO-*d*_6_) δ (ppm) = 9.71 (s, C–O**H**, 1H), 8.87 (s, -C–C**H** = N-, 1H), 7.51 (dd, *J* = 8.9, 3.2 Hz, -CF-C**H**-C–C = N-, 1H), 7.33 – 7.15 (m, -CF-C**H**-CH-COH- 2H), 6.97 (dd, *J* = 9, 4.5 Hz, = N–C-CH-C**H**-CH-COH-, 1H), 6.82 (ddd,* J* = 7.9, 2, 1 Hz, -COH-CH-CH-CF-,1H), 6.78 (dt,-COH-C**H**-CN-, 1H), 6.75 (ddd, = NC-C**H**-CH-1H); ^**13**^**C-NMR** δ(ppm) (151 MHz, DMSO-*d*_*6*_) δ 162.3 (C = N), 158.8, 157.0 (C-F), 154.7, 149.8, 130.8, 120.6, 120.0, 118.5, 117.5, 114.9, 112.6, 108.6 (Ar–C); **MS** m/z (%): 231.15 (100), 232.05(65.02).

##### 5-Fluoro-2-(((3-hydroxyphenyl)imino)methyl)phenol (M2)

Orange; mp: 145–146 ℃; Yield: 89%; **IR** υ_max_ (cm^−1^): 3062 (OH), 1620 (C = N); ^**1**^**H NMR** (400 MHz, DMSO-*d*_6_) δ (ppm) = 9.69 (s, = N–C-CH-CO**H**-, 1H), 8.92 (s, -C–C**H** = N-, 1H), 7.86–7.57 (m, -CH-C**H**-C–CH = N-, 1H), 7.24 (t,* J* = 8.0 Hz -CH-C**H**-CH-COH-, 1H), 6.92–6.64 (m, Ar–H, 5H); ^**13**^**C-NMR** δ (ppm) (151 MHz, DMSO-*d*_*6*_)δ 166.4 (C-F), 164.7, 162.9 (C = N), 158.9, 149.2, 135.3, 130.8, 116.9, 114.7, 112.5, 108.6, 107.3, 104.0 (Ar–C); **MS** m/z (%): 231.1 (100), 323.05 (38.26).

##### 2-(((4-Chlorophenyl)imino)methyl)-4-fluorophenol (M3)

Orange; mp: 124–125 ℃; Yield: 87%; **IR υ**_**max**_ (cm^−1^): 3065 (OH), 1615 (C = N); ^**1**^**H NMR** (600 MHz, Chloroform-*d*) δ (ppm) = 8.54 (s, C–C**H** = N-, 1H), 7.46–7.33 (m, -C**H**-CF-, 2H), 7.29–7.18 (m, -C**H**-CCl-, 2H), 7.14– 7.07 (m, = N–C-CH-, 2H), 6.98 (dd,* J* = 9.0, 4.4 Hz, -COH-C**H**-, 1H); ^**13**^**C-NMR** δ (ppm) = (151 MHz, CHLOROFORM-*d*) δ 161.9 (C = N), 157.3, 156.5 (C-F), 154.9, 146.7, 133.0, 129.7, 122.6, 120.7, 118.8, 118.6, 117.3, 117.2 (Ar–C); **MS** m/z (%): 249.05 (100), 250.05 (64.68).

##### 2-(((4-Chlorophenyl)imino)methyl)-5-fluorophenol (M4)

Gold; mp: 128–130 ℃; Yield: 90%; **IR υ**_**max**_ (cm^−1^): 3086 (OH), 1608 (C = N); ^**1**^**H NMR** (600 MHz, Chloroform-*d*) δ (ppm) = 8.56 (s, -C–C**H** = N-, 1H), 7.41–7.37 (m, = CH-C–C**H**-, 3H), 7.21 (d, *J* = 8.7 Hz, = N–C-C**H**-, 3H), 6.71 (dd, *J* = 10.6, 2.5 Hz, -CF-C**H**-CH-, 1H), 6.66 (td, *J* = 8.4, 2.5 Hz, -COH-C**H**-CF-, 1H); ^**13**^**C-NMR** δ (ppm) = (151 MHz, CHLOROFORM-*d*) δ 166.9 (C-F), 165.2, 163.5, 162.0 (C = N), 146.7, 134.3, 132.7, 129.7, 122.5, 116.0, 107.3, 107.2, 104.6 (Ar–C); **MS** m/z (%): 249.05 (100), 250.05 (56.93).

##### 1-(3-((5-Fluoro-2-hydroxybenzylidene)amino)phenyl)ethan-1-one (M5)

Yellow; mp: 130–132 ℃; Yield: 86%; **IR υ**_**max**_ (cm^−1^): 3341 (OH), 1620 (C = N), 1676 (C = O); ^**1**^**H NMR** (400 MHz, DMSO-*d*_6_) δ (ppm) = 8.99 (s, C–C**H** = N–C-, 1H), 7.95–7.84 (m, COCH_3_-C–C**H**-CH, 1H), 7.66 (d, *J* = 1.8 Hz, -CH-C**H**-CH, 1H), 7.62 (d, *J* = 7.5 Hz, = CH-C–C**H**-CF-, 1H), 7.55 (dd, *J* = 9.0, 3.2 Hz, = N–C-C**H**-C-COCH_3_, 1H), 7.35–7.26 (m, -CF-C**H**-CH-COH-, 1H), 7.18–7.07 (m, = N–C-C**H**-CH-, 1H), 7.00 (dd, *J* = 9.1, 4.6 Hz, -COH-C**H**-CH-, 1H), 2.64 (s, -COC**H**_3_, 3H). ^**13**^**C-NMR** δ (ppm) = (151 MHz, DMSO-*d*_*6*_)δ 198.2 (C = O), 163.2 (C = N), 156.90 (C-F), 149.43, 138.53, 130.39, 127.11, 126.64, 121.29, 118.61, 117.22, 113.18 (Ar–C), 27.41 (CH_3_); **MS** m/z (%): 257.15 (100), 258.05 (44.49).

##### 1-(3-((4-Fluoro-2-hydroxybenzylidene)amino)phenyl)ethan-1-one (M6)

Dark yellow; mp: 133–135 ℃; Yield: 90%; **IR υ**_**max**_ (cm^−1^): 3081 (OH), 1678 (C = O), 1619 (C = N);^**1**^**H NMR** (600 MHz, DMSO-*d*_6_) δ (ppm) = 9.04 (s, -C–C**H** = N-, 1H), 7.95 (t, *J* = 1.9 Hz, = N–C-C**H**-C-COH_3_, 1H), 7.89 (dt, *J* = 7.6, 1.4 Hz, -COCH3-C–C**H**-CH, 1H), 7.76 (dd, *J* = 8.5, 6.8 Hz, 1H), 7.67 (ddd, *J* = 7.9, 2.2, 1.1 Hz, 1H), 7.61 (t, *J* = 7.8 Hz, 1H), 6.90–6.79 (m, 1H), 2.64 (s, 3H); ^**13**^**C-NMR** δ (ppm) = (151 MHz, DMSO-*d*_*6*_) δ 198.2 (C = O), 166.5 (C-F), 164.2, 163.2 (C = N), 148.8, 138.6, 135.5, 130.4, 127.0, 126.7, 121.4, 117.0, 107.6, 104.2 (Ar–C), 27.5 (CH_3_); **MS** m/z (%): 257.1 (100), 258.05 (42.6).

##### 4-Fluoro-2-(((2-hydroxyphenyl)imino)methyl)phenol (M7)

Reddish orange; mp: 182–183 ℃; Yield: 92%; **IR υ**_**max**_ (cm^−1^): 3051 (OH), 1628 (C = N); ^**1**^**H NMR** δ (ppm) = (600 MHz, DMSO-*d*_6_) δ 9.77 (s, = N–C-CO**H**-, 1H), 8.95 (s, -C–C**H** = N-, 1H), 7.51 (dt, *J* = 9.0, 2.4 Hz, -C–C**H**-CF-, 1H), 7.34 (dt, *J* = 7.9, 1.3 Hz, -CF-C**H**-CH-, 1H), 7.30–7.18 (m, -CH-C**H**-CH-CH-, 1H), 7.19–7.10 (m, -CH-CH-CH-C**H**-, 1H), 7.14 (d, *J* = 1.6 Hz, 1H), 7.06–6.92 (m, = N–C-CH-C**H**-CH-, 2H), 6.91–6.83 (m, -COH-C**H**-, 1H); ^**13**^**C-NMR** δ (ppm) = (151 MHz, DMSO-*d*_*6*_) δ 160.7 (C = N), 157.4, 156.1 (C-F), 154.5, 151.8, 135.3, 128.9, 120.1, 120.0, 118.5, 117.5, 117.3, 117.1 (Ar–C)**; MS** m/z (%): 231.1 (96.94), 232.05 (35.21).

##### 5-Fluoro-2-(((2-hydroxyphenyl)imino)methyl)phenol (M8)

Gold; mp: 187–189 ℃; Yield: 87%; **IR υ**_**max**_ (cm^−1^): 3062 (OH), 1614 (C = N); ^**1**^**H NMR** (600 MHz, DMSO-*d*_6_) δ (ppm) = 9.86 (s, = N–C-CO**H** -,1H), 8.99 (s, -C–C**H** = N-, 1H), 7.65 (dd, *J* = 8.6, 7.0 Hz, = CH-C–C**H**-, 1H), 7.39 (dd, *J* = 8.0, 1.6 Hz, -COH-CH-C**H**-, 1H), 7.13 (ddd, *J* = 8.1, 7.3, 1.6 Hz, = N–C-C**H**-, 1H), 6.97 (dd, *J* = 8.1, 1.4 Hz, -COH-C**H**-, 1H), 6.88 (td, *J* = 7.6, 1.4 Hz, -COH-C**H**-CF-, 1H), 6.83–6.58 (m, = N–C-CH-C**H**-CH-, 2H); ^**13**^**C-NMR** δ (ppm) = (151 MHz, DMSO-*d*_*6*_) δ 166.5 (C-F), 165.3, 164.9, 160.8 (C = N), 151.4, 135.2, 134.2, 128.7, 120.2, 119.8, 117.0, 106.6, 104.3 (Ar–C); **MS** m/z (%): 231.1 (100), 232.05 (37.06).

##### 2,2'-((1*E*,1'*E*)-(1,2-phenylenebis(azaneylylidene))bis(methaneylylidene))bis(4-fluorophenol) (M9)

Yellow; mp: 129–131 ℃; Yield: 88%; **IR υ**_**max**_ (cm^−1^): 3357 (OH), 1614 (C = N); ^**1**^**H NMR** (600 MHz, Chloroform-*d*) δ (ppm) = 8.57 (s, -C-N = C**H**-C, 2H), 7.4–7.34 (m, -CF-C**H**-C–CH = , 2H), 7.28–7.22 (m, N–C-CH-C**H**-, 2H), 7.13–7.06 (m, = N–C-C**H**-, 4H), 7.01 (dd, *J* = 8.9, 4.5 Hz, = CH-C-COH-C**H**-, 2H); ^**13**^**C-NMR** δ (ppm) = (151 MHz, CHLOROFORM-*d*) δ 162.7 (2C = N), 157.6, 156.4 (2C-F), 154.8, 142.3, 128.2, 120.8, 120.6, 119.7, 118.9, 118.88, 118.83, 118.78, 117.2, 117.1 (Ar–C); MS m/z (%): 352(13), 350 (50).

##### 6,6'-((1*E*,1'*E*)-(1,2-phenylenebis(azaneylylidene))bis(methaneylylidene))bis(3-fluorophenol) (M10)

Orange; mp: 131–133 ℃; Yield: 89%; **IR υ**_**max**_ (cm^−1^): 3387 (OH), 1608 (C = N); ^**1**^**H NMR** (600 MHz, DMSO-*d*_6_) δ (ppm) = 8.95 (s,-C–C**H** = N-, 2H), 7.84–7.67 (m, = CH-C–C**H**-, 2H), 7.48 (dd, *J* = 6.0, 3.4 Hz, = N–C-CH-C**H**-, 2H), 7.40 (dd, *J* = 5.9, 3.4 Hz, = N–C-C**H**-, 2H), 6.91–6.71 (m, -CF-C**H**-, 4H); ^**13**^**C-NMR** (151 MHz, DMSO-*d*_*6*_) δ (ppm) = 166.6 (2C-F), 164.9, 163.6, 163.5 (2C = N), 142.2, 135.24, 135.16, 128.4, 120.2, 117.2, 107.4, 107.2, 104.3, 104.1 (Ar–C); **MS** m/z (%): 352.1 (42.07), 353.05 (19.51).

### TGA

The TGA samples were analysed by Thermogravimetric Analyzer (Pyris 6 TGA), PerkinElmer for around 32 min each and the heat was kept from 30 to 700 °C under nitrogen gas at a rate: 20 degrees/minute.

### Mercury adsorption testing

To visualize the chelation properties, a UV–Vis spectrophotometer was employed. Initially, stock solutions of mercury (II) chloride and the Schiff bases were prepared at a concentration of 1000 ppm in volumetric flasks. Specifically, 100 mg of mercury (II) chloride was dissolved in 100 mL of distilled water, while 100 mg of each Schiff base was dissolved in 100 mL of warm ethanol and stirred for 15 min to ensure complete dissolution. The UV–Vis spectrophotometer was configured to measure absorbance over a wavelength range of 200 to 700 nm using a Shimadzu UV-2600i UV–Vis spectrometer, integrated with LabSolutions UV–Vis software. For the chelation study, the Schiff bases and mercury chloride solutions were subsequently diluted to final concentrations of 10 ppm and 1 ppm, respectively.

### Calculating the adsorption efficiency

To measure the ability of the fluorinated Schiff bases to adsorb mercury with the aid of a UV–Vis spectrometer, the process begins with establishing a calibration curve of Schiff bases at different concentrations. After preparing the calibration curve, the adsorption testing is conducted using 10 ppm of different Schiff bases and 1 ppm of mercury (II) chloride. For each adsorption test, 3 ml of the Schiff base solution at 10 ppm is added to the cuvette and tested through the UV–Vis spectrometer to observe the absorbance peaks. Then, 25 µl of mercury is added to the same cuvette containing the Schiff base and the spectrum is measured again. This procedure is repeated and carried out until the trend seen by the peak has changed. The concentration of mercury required to disrupt the chelation was measured, hinting at the idea of no more adsorption by the Schiff base. The concentration of mercury adsorbed was then measured and evaluated among the Schiff bases. Finally, a matrix is created to summarize the Schiff bases' effectiveness in adsorbing mercury, utilizing an equation derived from wastewater studies to quantify the adsorption efficiency. [49]

$$\%adsorption=\frac{\left(Co-Ce\right)}{Co}\times 100\%$$

where Co is the initial concentration of Schiff base used and Ce is the equilibrium concentration of Schiff base, defined as the concentration at which the trend in the UV–Vis spectrum changes, indicating the disruption of chelation.

### Schiff base stability testing

To test the stability, a 1cm quartz cuvette was used, 3ml of 10 ppm Schiff base was added and mixed with 1ppm of 25µl of mercury chloride and the absorbance peak was measured, the same mixture in the cuvette was measured every 5 min for 30 min to observe any difference in the absorption peak leading to degeneration of the compound and inefficiency to act as an adsorbent for mercury. To assess the stability of the Schiff bases, a 1 cm quartz cuvette was utilized. A mixture of 3 mL of 10 ppm Schiff base was combined with 25 µL of 1 ppm mercury (II) chloride, and the absorbance peak was recorded. This mixture was subsequently measured every 5 min over a 30-min period to monitor any changes in the absorption peak. Such changes would indicate potential degradation of the compound and its diminishing efficacy as an adsorbent for mercury.

## Results and discussion

### Synthesis of Schiff base

**Scheme 1:**
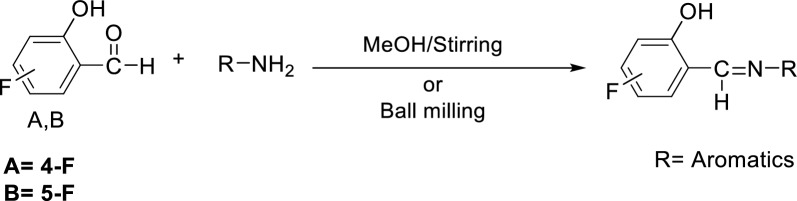
General synthesis of fluorine-based Schiff base ligands

In this study, the synthesized fluorinated Schiff bases were prepared using ball milling and conventional reflux (Scheme 1). It was seen from the results obtained that the Schiff bases isolated from the ball milling technique have the same physical properties compared with those isolated by the conventional method of preparation. When we make a comparison between yield and time taken for the preparation of these ligands, the results show that the synthesis of these compounds using the ball milling technique is faster and more productive (Table 1). The ball milling method achieved the desired products in a significantly shorter time, with the shortest reaction time being 5 min for **M1**, **M2**, **M3**, and **M4**. Ball milling also resulted in higher yields for all reactions from **M1** to **M10**, with the highest yield observed for **M7** at 92%. Importantly, ball milling avoids the need for toxic solvents like methanol, which were required in the conventional method. In ball milling, Solvents were used only for recrystallization, if necessary.

**Table 1:**
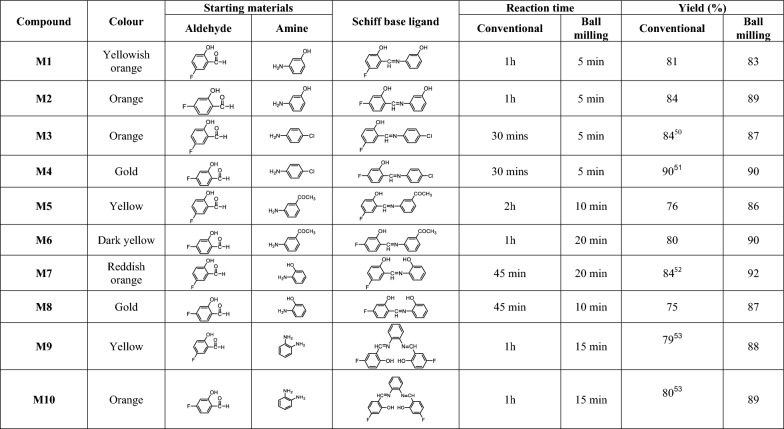
Colour and comparison between reaction time and yields of the synthesized Schiff base ligands

### FTIR, NMR and mass analysis

**Fig. 2:**
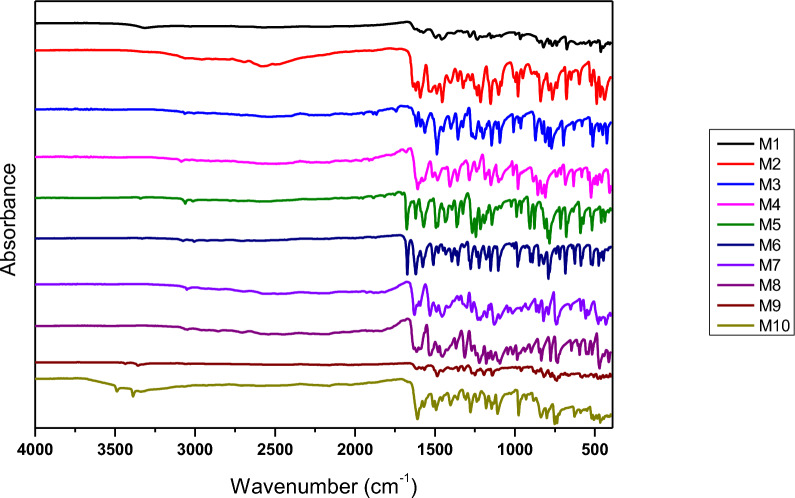
FTIR spectrum of Schiff bases M1-M10

FTIR spectra of all Schiff bases are presented in Fig. 2. FTIR analysis confirmed the successful synthesis of Schiff bases, highlighted by the characteristic imine (C=N) vibrational peaks observed in all compounds within the range of 1573–1620 cm⁻^1^, consistent with literature values. This validates the condensation reaction between the starting materials (aldehyde and amine groups), leading to the formation of the target compounds. The O–H vibrational peaks, indicative of hydroxyl groups, were partially diminished and shifted in most compounds, suggesting reduced availability of free hydroxyl groups due to intramolecular or intermolecular hydrogen bonding. Compound **M2** displayed neither O–H nor aromatic C–H peaks, indicating pronounced hydrogen bonding or significant electronic structure alterations. Furthermore, no detectable C=O peaks of aldehydes were observed, confirming the complete consumption of aldehyde starting materials. Overall, the FTIR results substantiate the formation of Schiff bases, as evidenced by the presence of imine peaks, the absence of aldehyde C=O peaks, and structural modifications reflected in diminished O–H and aromatic C–H vibrations.

The NMR data for the synthesized compounds (**M1-M10**) clearly demonstrate their structural properties, including distinctive signals for hydroxyl (OH), imine (CH = N), carbonyl (C=O), and fluoro-substituted aromatic systems. The chemical shift variations, notably for C=N and C-F signals, reveal the differentiation between two sets of compounds based on their starting materials: 5-fluoro-2-hydroxybenzaldehyde (**M1, M3, M5, M7, M9**) and 4-fluoro-2-hydroxybenzaldehyde (**M2, M4, M6, M8, M10**). In the ^1^H NMR spectra of all compounds, the presence of a hydroxyl (OH) signal appearing between 9.69–9.86 ppm, confirms the presence of a phenolic moiety. The CH=N proton signal, observed between 8.54–9.04 ppm, is consistent across all compounds, indicating the formation of Schiff bases. Notably, **M5** and **M6** exhibit an additional methyl (CH_3_) peak at 2.64 ppm, which confirms the presence of COCH_3_ in the structure. In the ^13^C NMR spectra, the C=N carbon signal consistently appears between 160.7–163.2 ppm, further validating the presence of imine functionalities. The C-F signals, ranging from 156.1 to 166.9 ppm, exhibit slight variations based on the position of the fluorine atom in the starting aldehyde, suggesting that the electronic environment around the fluorinated aromatic ring affects the de-shielding effect. For **M5** and **M6**, the strong C=O signal at 198.2 ppm confirms the presence of a ketone functional group, differentiating these two from the other Schiff bases. Additionally, the methyl carbon (CH_3_) in **M5** and **M6** appears at 27.41 ppm, in agreement with their ^1^H NMR data.

In comparison to compounds formed from 4-fluoro-2-hydroxybenzaldehyde (**M2, M4, M6, M8, M10**), compounds derived from 5-fluoro-2-hydroxybenzaldehyde (**M1, M3, M5, M7, M9**) exhibit somewhat larger up-field changes in C=N (161.9–163.2 ppm). This change might be because fluorine has various effects on delocalization inside the aromatic system depending on whether it is at the para (4-fluoro) or meta (5-fluoro) position, which draws electrons differently.

The mass spectrum of compound **M1** showed a base peak at m/z 231.15 with 100% intensity, indicating it as the most stable fragment, along with a molecular ion peak (M^+^) at m/z 232.05 with 65.02% intensity, confirming the molecular weight of the compound. Similarly, **M2** exhibited a base peak at m/z 231.1 (100%), with its molecular ion peak at m/z 232.05 (38.26%), showing a similar fragmentation pattern. For **M3** and **M4**, the base peak was observed at m/z 249.05 (100%), with the corresponding molecular ion peak (M^+^) at m/z 250.05, with intensities of 64.68% and 56.93%, respectively, indicating a stable molecular core. Compounds **M5** and M6 showed base peaks at m/z 257.15 and 257.1 (100%), with molecular ion peaks at m/z 258.05 (44.49% and 42.6%), suggesting a heavier molecular structure with isotopic distribution.

For **M7** and **M8**, the base peak appeared at m/z 231.1, with molecular ion peaks at m/z 232.05 (35.21% for M7) and 323.05 (37.06% for M8), possibly indicating different fragmentation pathways or adduct formation in M8. Finally, M10 displayed a base peak at m/z 352.1 (42.07%), with a molecular ion peak (M^+^) at m/z 353.05 (19.51%), confirming a higher molecular weight structure.

### TGA analysis

**Fig. 3:**
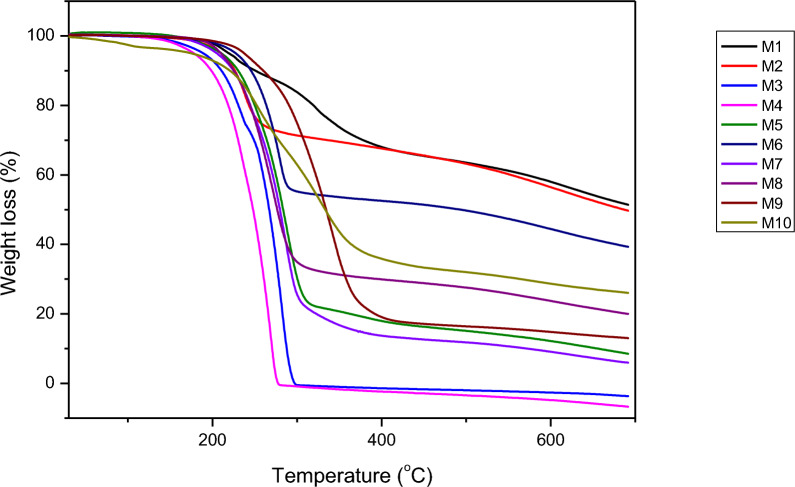
Thermogravimetric analysis (TGA) curve of the Schiff base ligands M1-M10, recorded under a nitrogen atmosphere with a heating rate of 20°C/min over a temperature range of 30–700 °C

The thermal properties of the Schiff base compounds were analysed using a Thermogravimetric Analyzer (Pyris 6 TGA, PerkinElmer) under a nitrogen atmosphere to maintain an inert environment. The analysis was conducted at a controlled heating rate of 20°C/min over a temperature range of 30–700°C. The resulting TGA curves provided insights into the thermal stability and decomposition patterns of the synthesized Schiff bases (Fig. 3).

The Schiff base compounds exhibited significant thermal stability up to approximately 200°C, as indicated by the absence of notable weight loss in this range, reflecting their structural robustness under moderate heating conditions. A significant weight loss occurred around 300°C, attributed to the thermal cleavage of the imine (C = N) and phenol (OH) functional groups, marking the initial breakdown of the core molecular structure. Beyond 300°C, a gradual decrease in weight was observed, likely due to the loss of aromatic fragments and the formation of smaller, thermally resistant fluorinated aromatic moieties. Notably, the residues of most compounds, except **M3** and **M4**, suggest the presence of stable fluorinated aromatic fragments, highlighting the distinctive stability of these Schiff base derivatives. Table2**,** summarizes the decomposition temperature ranges along with the corresponding weight loss percentages for each Schiff base.

**Table 2:** Thermogravimetric Analysis (TGA) Data of Schiff Base Ligands M1–M10, Indicating Decomposition Temperature Ranges and Corresponding Weight Loss %

Compound	Decomposition Temperature range °C	Weight loss %
M1	≈ 190°C–370°C	28
M2	≈ 191°C–268°C	25
M3	≈ 168°C–289°C	97
M4	≈ 150°C–277°C	98
M5	≈ 173°C–312°C	75
M6	≈ 213°C–291°C	41
M7	≈ 190°C–340°C	80
M8	≈ 194°C–310°C	65
M9	≈ 221°C–400°C	78
M10	≈ 197°C–380°C	55

TGA was primarily conducted to assess the thermal stability of the Schiff bases, which is a critical parameter for their potential applications in metal adsorption. Since metal adsorption requires structurally stability, and the processes often occur under varying environmental conditions, including elevated temperatures, understanding the thermal behaviour of these compounds is essential. The ability of these Schiff bases to maintain structural integrity at high temperatures suggests that they can be tested in harsher environments where thermal resistance is crucial, such as industrial wastewater treatment. This analysis also reinforces their potential as efficient mercury adsorbents, ensuring their stability during adsorption processes. Additionally, the TGA results confirm the purity of the synthesized Schiff bases, as the absence of additional weight-loss events indicates minimal impurities.

An interesting trend observed in the thermal stability data was the difference between para- and meta-substituted Schiff bases. Compounds with substituents in the para position exhibited slightly higher thermal stability compared to their meta counterparts. This enhanced stability may be attributed to the greater potential for hydrogen bonding interactions between the hydrogen and fluorine atoms in the para-substituted compounds, which reinforces the molecular framework against thermal degradation.

### Mercury adsorption testing

A Shimadzu UV-2600i UV–Vis spectrometer, integrated with LabSolutions UV–Vis software, was utilized to measure the absorbance spectra of Schiff base ligands within the 200–700 nm wavelength range. This analysis was conducted to detect variations in absorbance profiles resulting from the interaction of Schiff bases with mercury ions, indicating chelation and adsorption efficacy. Schiff bases are well-recognized for their chelating properties, attributed to the availability of lone electron pairs on functional groups such as nitrogen, hydroxyl, and carbonyl. These functional groups can form coordination bonds with transition metals, including mercury, due to their electron-donating properties.

Compounds selected for this study, labelled **M6**, **M7**, **M8**, and **M9**, possess structural variations such as dual hydroxyl groups or combinations of hydroxyl and carbonyl groups, which were hypothesized to enhance mercury chelation. Notably, no previous studies have reported using these specific compounds for mercury chelation, making this investigation a novel exploration of their binding capabilities. Each experimental procedure commenced with generating a calibration curve for each compound, providing a reference for analysing the changes in absorbance associated with mercury adsorption. The concentration used to calculate the calibration curve was 1, 10, 20, 50 and 100 ppm. Using these concentrations, the molar absorptivity was found 5965, 15,194, 12,710 and 24,655, respectively for **M6**, **M7**, **M8** and **M9**.

**Fig. 4:**
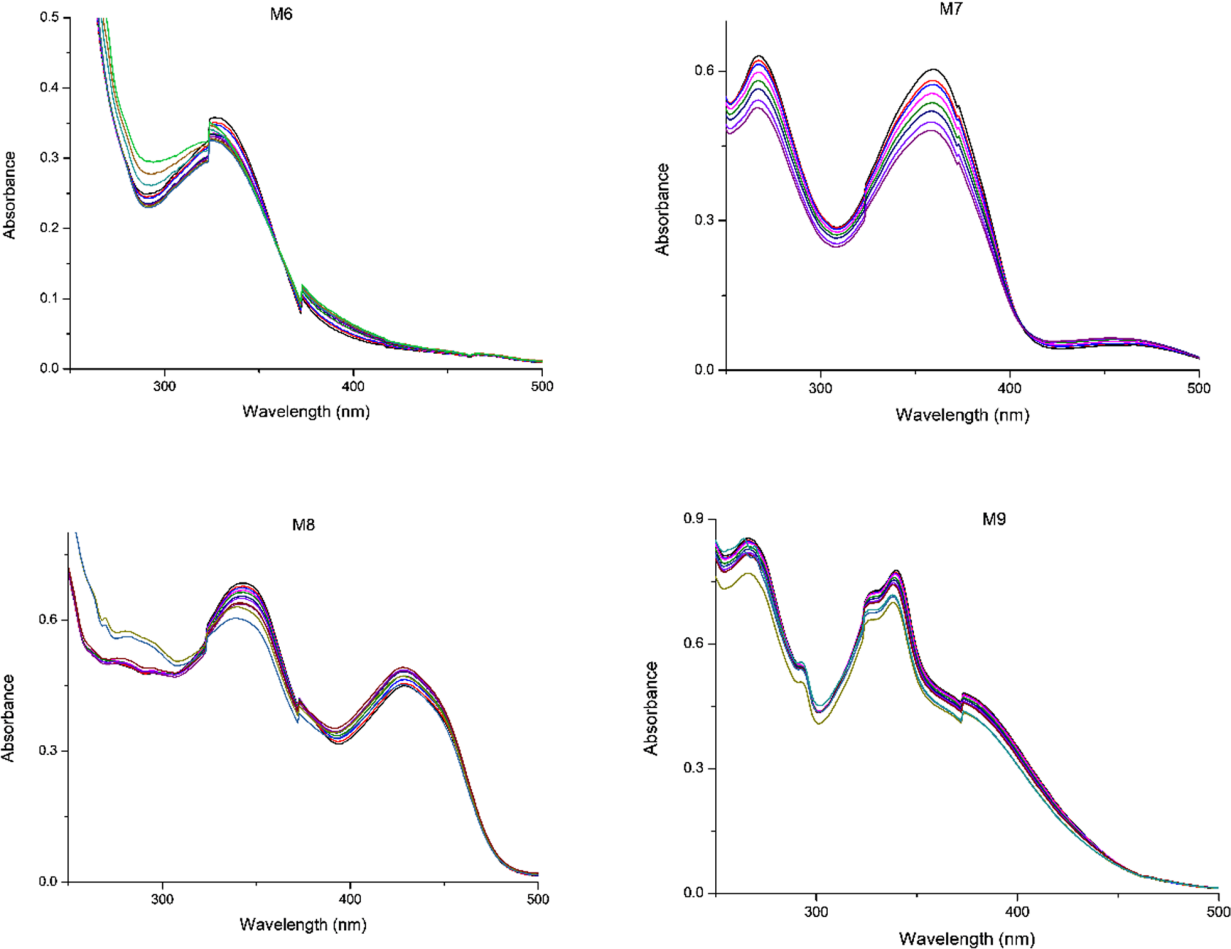
UV–Vis absorbance spectrum of Schiff bases (M6-M9) in ethanol at a concentration of 10 ppm

After the determination of molar absorptivity, each Schiff base ligand was subjected to chelation testing, guided by changes in absorbance peaks as shown in Fig. 4. The experiments were initiated with 10 ppm solutions of Schiff base and 1 ppm mercury (II) chloride solution. Initially, 3 mL of the Schiff base solution alone was measured to establish a baseline for comparison and to observe any changes that occurred with the addition of mercury ions. Mercury was introduced incrementally by adding 25 µL of the mercury solution to the same cuvette containing 3 mL of the Schiff base solution. The addition of mercury solution was continued until significant changes in peak patterns were observed.

A common trend across all Schiff bases was a decrease in the intensity of absorbance at the peak maxima after the addition of mercury, indicating a hypochromic shift. This phenomenon is consistent with the formation of chelating bonds and may be attributed to a reduction in π-electron density and increased structural rigidity upon interaction with mercury ions [54]. The hypochromic effect provides strong evidence for chelation between Schiff bases and mercury.

Among the tested Schiff bases, **M7** exhibited the best performance, likely due to its structural advantage of having two OH groups positioned to effectively coordinate with mercury ions, giving stronger chelation, also the presence of fluorine enhances the affinity for Hg^2^⁺ ions by increasing the electron density at coordination sites and modulating the polarity of the molecule, thereby improving metal-binding interactions. **M9**, although possessing two nitrogen lone pairs and a hydroxyl group, was outperformed by **M7**. This is likely due to the bulky structure in **M9**, which introduces steric hindrance, reducing its efficiency of chelation with mercury ions.

**M6** and **M8** demonstrated comparable results, with **M6** performing slightly better. **M6**, which contains only one hydroxyl group, was less effective than **M7** as anticipated. However, the poor performance of **M8**, despite its structural similarity to **M7**, was notable. The primary difference lies in the position of the fluorine atom: **M7** has the fluorine substituent at the para position, while **M8** has it at the meta position. The meta position is known to exert stronger electron-withdrawing effects, which can inhibit the electron-donating capability of the hydroxyl group, thereby reducing the efficiency of chelation with mercury ions.

### Schiff base efficacy

The effectiveness of Schiff bases in chelating mercury ions is studied. This is carried out using a UV–Vis spectrometer. The experiments were conducted until noticeable changes in the absorbance maxima trends were observed, either through the appearance of a new peak or the absence of a decrease in absorbance intensity. The critical points at which this trend changed occurred were recorded as follows for the Schiff base compounds **M6**, **M7**, **M8**, and **M9**: 150 µL, 600 µL, 125 µL, and 325 µL of mercury, respectively, which indicates saturation or significant interaction.

To further analyze the data, interpolation was used to determine the amount of each Schiff base compound required to chelate mercury ions. This is calculated with the aid of the absorbance found at the critical point in conjunction with the molar absorptivity of each compound. We can determine the percentage of each Schiff base used for adsorption by comparing the initial concentration of Schiff bases used in the experiments with the final concentration determined through mercury adsorption tests (Table 3).

**Table 3:** Calculations for the efficacy of Schiff bases

Schiff base	Original Concentration of Schiff base (M)	Concentration of Schiff base used in adsorption (M)	Percentage of Schiff base used to adsorb (%)
M6	6.00E-05	5.55E-05	7.50
M7	3.97E-05	3.27E-05	17.6
M8	5.38E-05	5.15E-05	4.50
M9	3.15E-05	2.99E-05	5.15

**Fig. 5:**
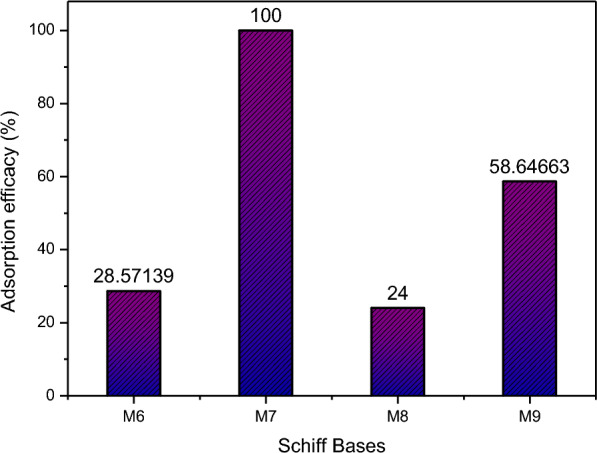
Adsorption Efficacy of Schiff Base Compounds (M6, M7, M8, and M9) for Mercury Ions at Critical Points

In order to find the efficacy of Schiff bases, **M7**, which demonstrated the highest adsorption capacity, was selected as the reference compound. The effectiveness of remaining Schiff bases was assessed relative to **M7** to establish a framework for comparing their performance as mercury adsorbents (Fig. 5).

### Degradation assessment

**Fig. 6:**
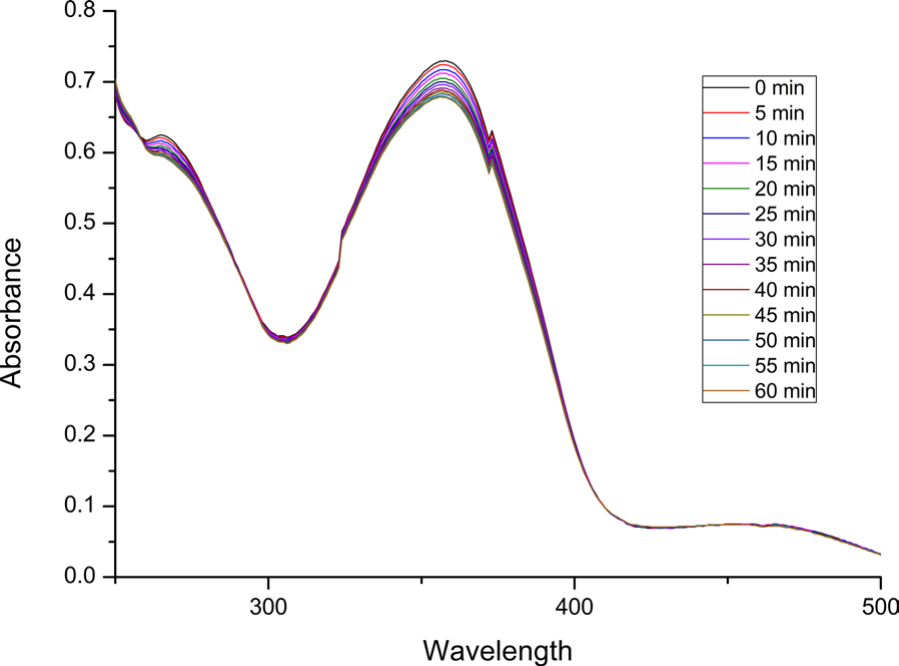
UV–Vis absorbance spectrum of Schiff base M7 (10 ppm in solution) during its stability evaluation over a 60-min reaction with mercury (II) chloride (1 ppm)

Given the superior performance of **M7** in UV–Vis absorption, its stability was evaluated for further study (Fig. 6). A solution of the Schiff base **M7** (3 mL, 10 ppm) was prepared and mixed with mercury (II) chloride solution (25 µL, 1 ppm). The mixture was allowed to react for 60 min, during which absorbance measurements were recorded using a UV–Vis spectrometer at 5-min intervals. A gradual decrease in absorbance intensity was observed over time, indicating the chelation of mercury ions by the Schiff base **M7**. This reduction in absorbance is attributed to a decrease in the electron density within the **M7** molecule as it binds with mercury ions, aligning with the expected chelation mechanism and confirming the interaction between the Schiff base and mercury ions. As the reaction progressed, the rate of change in absorbance between successive intervals gradually diminished, suggesting that the system was nearing saturation of the adsorbent. By the end of the 60-min period, no further reduction in absorbance was observed, indicating that the chelation process had reached equilibrium, with all available binding sites on the Schiff base **M7** likely occupied by mercury ions.

The UV–Vis spectrum showed no new peaks or significant spectral changes during the experiment, indicating that Schiff base **M7** remained structurally intact throughout the chelation process. This absence of further changes after 60 min confirmed the stability of **M7** under experimental conditions, and these findings highlight **M7**’s potential as a stable and effective chelating agent for Hg^2+^ ions.

## Conclusion

This study successfully demonstrated the synthesis of ten fluorinated Schiff bases via a ball milling approach, establishing it as a highly efficient, solvent-free, and environmentally sustainable alternative to conventional methods. The mechanochemical process significantly enhanced all reaction efficiency, with some Schiff bases completed in under five minutes and yields reaching up to 92%, far surpassing those achieved by refluxing protocols. The mercury adsorption performance of some selected synthesized compounds was thoroughly evaluated, with M7 exhibiting the highest adsorption capability (17.6%), underscoring its potential as an effective adsorbent for environmental remediation. With the growing concern over heavy metal contamination, these findings emphasize the value of ball milling as a sustainable technique for developing effective adsorbents. Additionally, we are currently conducting toxicity studies on these compounds, alongside further investigations into their potential anticancer and antimicrobial properties. Future research will aim to optimize their structures to enhance their adsorption efficiency while expanding their applications in both environmental and biomedical fields.

## Supplementary Information

Supplementary material 1.

## Data Availability

We have presented all our main data in the form of tables, figures, and also in the supplementary information file.
