# Supplementary material for: Efficient mechanochemical synthesis of new fluorinated Schiff bases: a solvent-free, alternative to conventional method with mercury adsorption properties
Source: BMC Chem. 2025 Jul 4;19(1):201. doi: 10.1186/s13065-025-01552-9 (PMC12231667; doi:10.1186/s13065-025-01552-9)
Supplement: Supplementary file 1 — Supplementary material 1. [file 13065_2025_1552_MOESM1_ESM.pdf]

Supporting information

Efficient Mechanochemical Synthesis of New Fluorinated Schiff Bases: A Solvent-Free, Alternative to Conventional Method with Mercury Adsorption Properties

Mirza T. Baig[1], Mariam T. Sayed[1], Reem Aledamat[1], Sumyah Hassan[1], Alaa AlReyashi[1], Naheed Sidiq[1], Siham Y. Al-Qaradawi[1], and Mohamed F. Mady[1*]

[1]*Department of Chemistry and Earth Sciences, College of Arts and Science, Qatar University, Doha, Qatar*

*Corresponding author: M.F. Mady

E-mail address: mmady@qu.edu.qa

Table of Contents

Figure S1. FTIR spectrum of M1	S3
Figure S2. ^{1}H NMR spectrum of M1	S4
Figure S3. ^{13}C NMR spectrum of M1	S4
Figure S4. Mass spectrum of M1	S5
Figure S5. FTIR spectrum of M2	S5
Figure S6. ^{1}H NMR spectrum of M2	S6
Figure S7. ^{13}C NMR spectrum of M2	S6
Figure S8. Mass spectrum of M2	S7
Figure S9. FTIR spectrum of M3	S7
Figure S10. ^{1}H NMR spectrum of M3	S8
Figure S11. ^{13}C NMR spectrum of M3	S8
Figure S12. Mass spectrum of M3	S9
Figure S13. FTIR spectrum of M4	S9
Figure S14. ^{1}H NMR spectrum of M4	S10
Figure S15. ^{13}C NMR spectrum of M4	S10
Figure S16. Mass spectrum of M4	S11
Figure S17. FTIR spectrum of M5	S11

Figure S18. ^{1}H NMR spectrum of M5	S12
Figure S19. Mass spectrum of M5	S12
Figure S20. FTIR spectrum of M6	S13
Figure S21. ^{1}H NMR spectrum of M6	S13
Figure S22. ^{13}C NMR spectrum of M6	S14
Figure S23. Mass spectrum of M6	S14
Figure S24. FTIR spectrum of M7	S15
Figure S25. ^{1}H NMR spectrum of M7	S16
Figure S26. ^{13}C NMR spectrum of M7	S16
Figure S27. Mass spectrum of M7	S17
Figure S28. FTIR spectrum of M8	S17
Figure S29. ^{1}H NMR spectrum of M8	S18
Figure S30. ^{13}C NMR spectrum of M8	S18
Figure S31. Mass spectrum of M8	S19
Figure S32. FTIR spectrum of M9	S19
Figure S33. ^{1}H NMR spectrum of M9	S20
Figure S34. ^{13}C NMR spectrum of M9	S20
Figure S35. Mass spectrum of M9	S21
Figure S36. FTIR spectrum of M10	S21
Figure S37. ^{1}H NMR spectrum of M10	S22
Figure S38. ^{13}C NMR spectrum of M10	S22
Figure S39. Mass spectrum of M10	S23

Number of Pages: 29
Number of Figures: 29

1) 4-Fluoro-2-(((3-hydroxyphenyl)imino)methyl)phenol (M1)

Yellowish orange; mp: 143-145 °C; Yield: 83%; **IR** υ_{max} (cm^{-1}): 3315 (OH), 1625 (C=N); **^{1}H NMR** (600 MHz, DMSO-d_6) δ(ppm)= 9.71 (s, C-O**H**, 1H), 8.87 (s, -C-C**H**=N-, 1H), 7.51 (dd, *J*= 8.9, 3.2 Hz, -CF-C**H**-C-C=N-, 1H), 7.33 – 7.15 (m, -CF-C**H**-CH-COH- 2H), 6.97 (dd, *J*=

9, 4.5 Hz ,=N-C-CH-C**H**-CH-COH-, 1H), 6.82 (ddd, *J*= 7.9, 2, 1 Hz, -COH-CH-CH-CF- ,1H), 6.78 (dt,-COH-C**H**-CN-, 1H), 6.75 (ddd, =NC-C**H**-CH-1H); **^{13}C-NMR** (151 MHz, DMSO-d_6) δ 162.3 (C=N), 158.8, 157.0 (C-F), 154.7, 149.8, 130.8, 120.6, 120.0, 118.5, 117.5, 114.9, 112.6, 108.6 (Ar-C); **MS** m/z (%): 231.15 (100), 232.05(65.02).

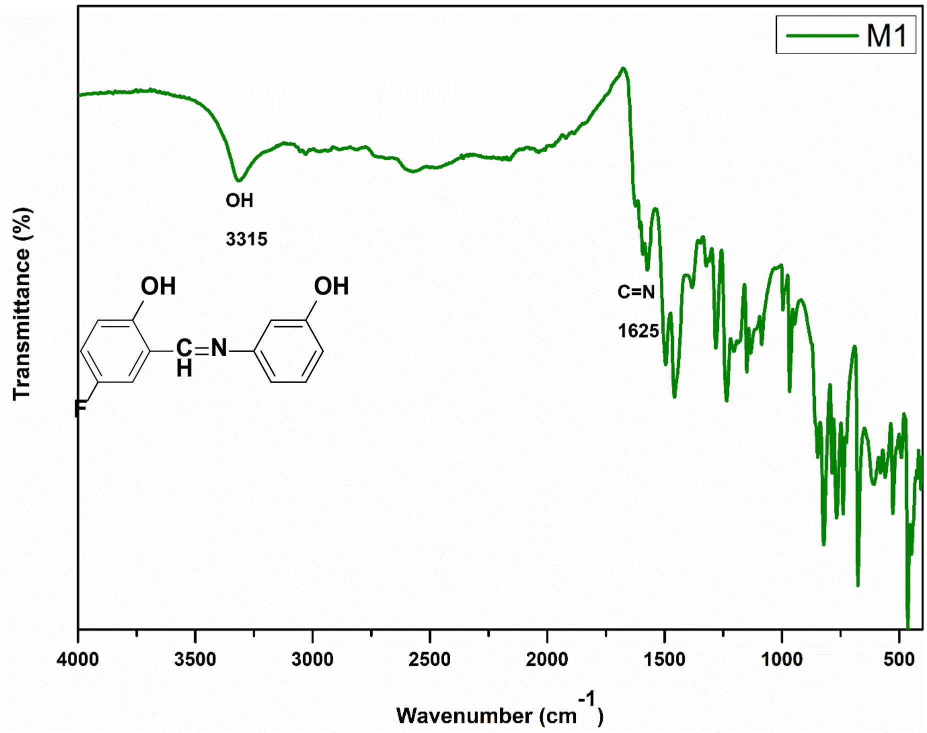

Figure S1. FTIR spectrum of M1

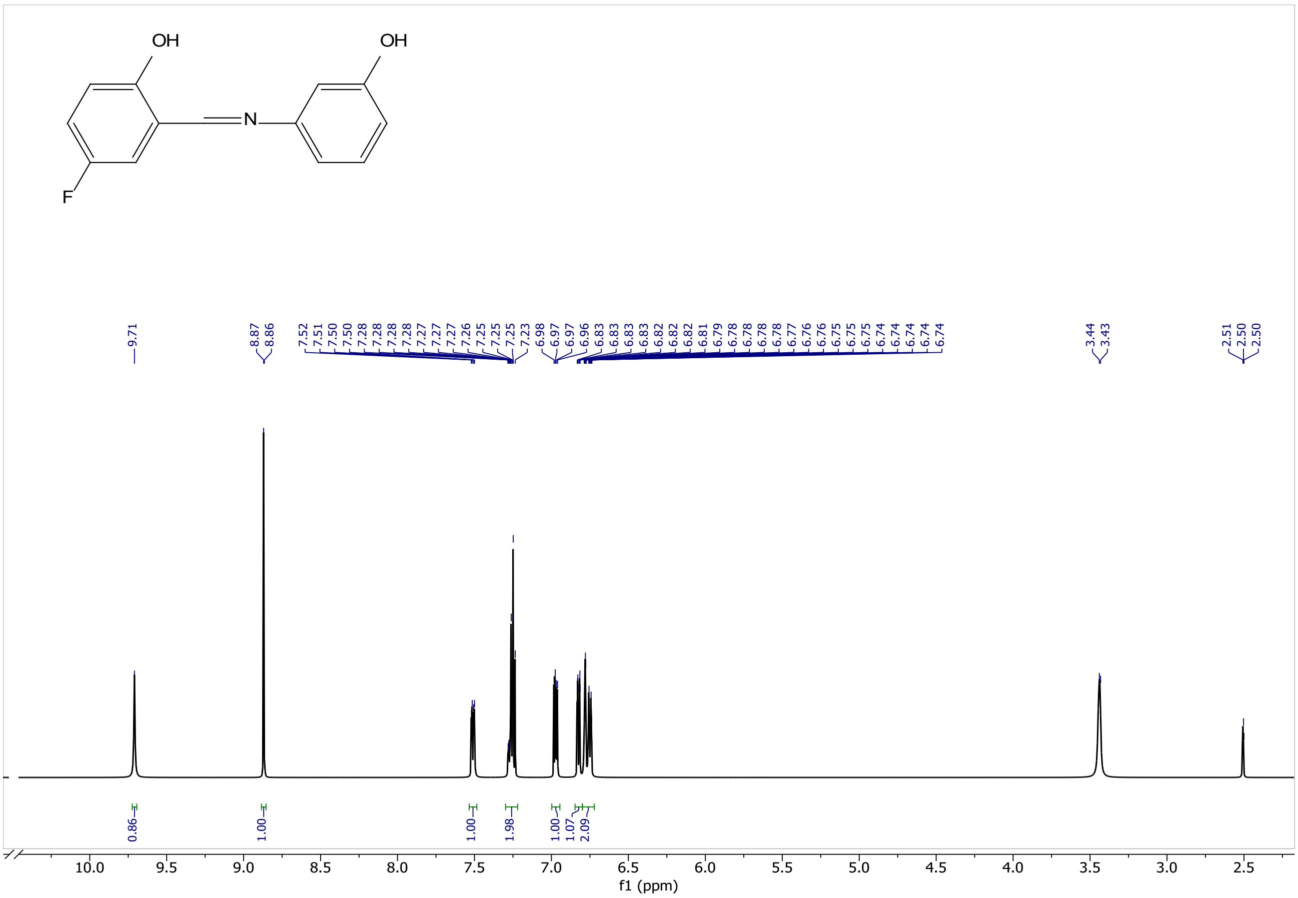

Figure S2. ^{1}H NMR spectrum of M1

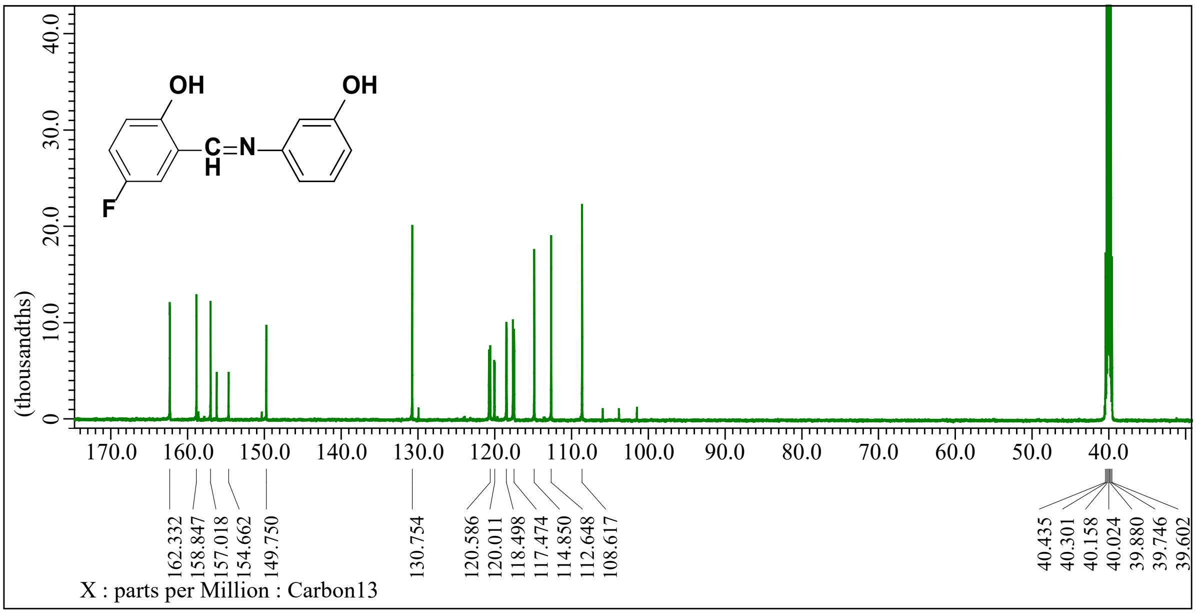

Figure S3. ^{13}C NMR spectrum of M1

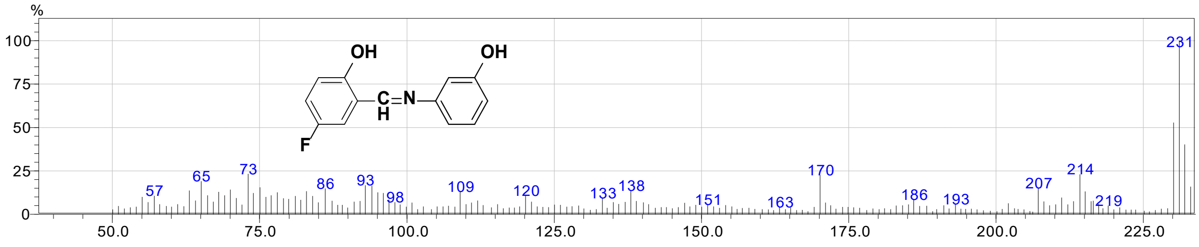

Figure S4. Mass spectrum of M1

2) 5-Fluoro-2-(((3-hydroxyphenyl)imino)methyl)phenol (M2)

Orange; mp: 145-146 °C; Yield: 89%; **IR** υ_{max} (cm^{-1}): 3062 (OH), 1620 (C=N); **^{1}H NMR** (400 MHz, DMSO-d_6) δ (ppm) = 9.69 (s, =N-C-CH-CO**H**-, 1H), 8.92 (s, -C-C**H**=N-, 1H), 7.86 – 7.57 (m, -CH-C**H**-C-CH=N-, 1H), 7.24 (t, *J*= 8.0 Hz -CH-C**H**-CH-COH-, 1H), 6.92 – 6.64 (m, Ar-H, 5H); **^{13}C-NMR** (151 MHz, DMSO-d_6)δ 166.4 (C-F), 164.7, 162.9 (C=N), 158.9, 149.2, 135.3, 130.8, 116.9, 114.7, 112.5, 108.6, 107.3, 104.0 (Ar-C); **MS** m/z (%): 231.1 (100), 323.05 (38.26).

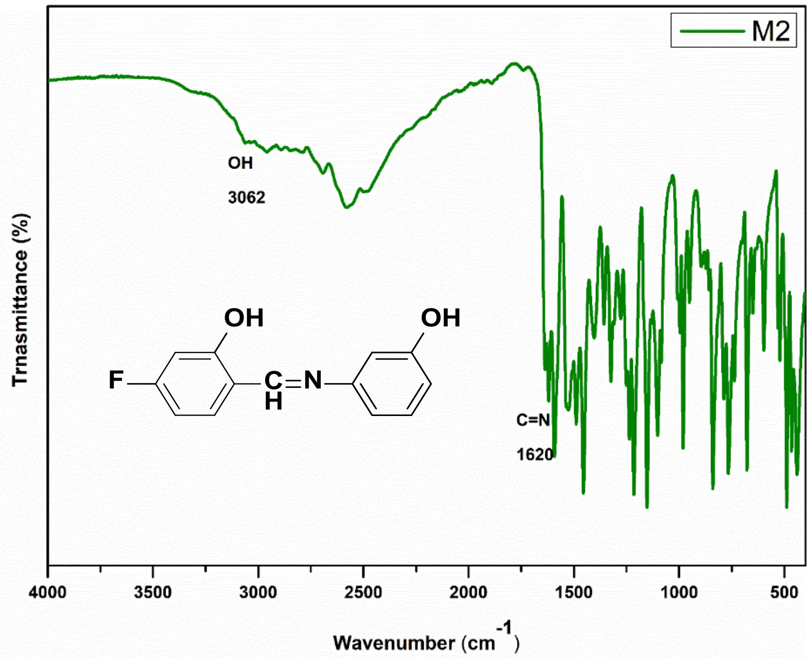

Figure S5. FTIR spectrum of M2

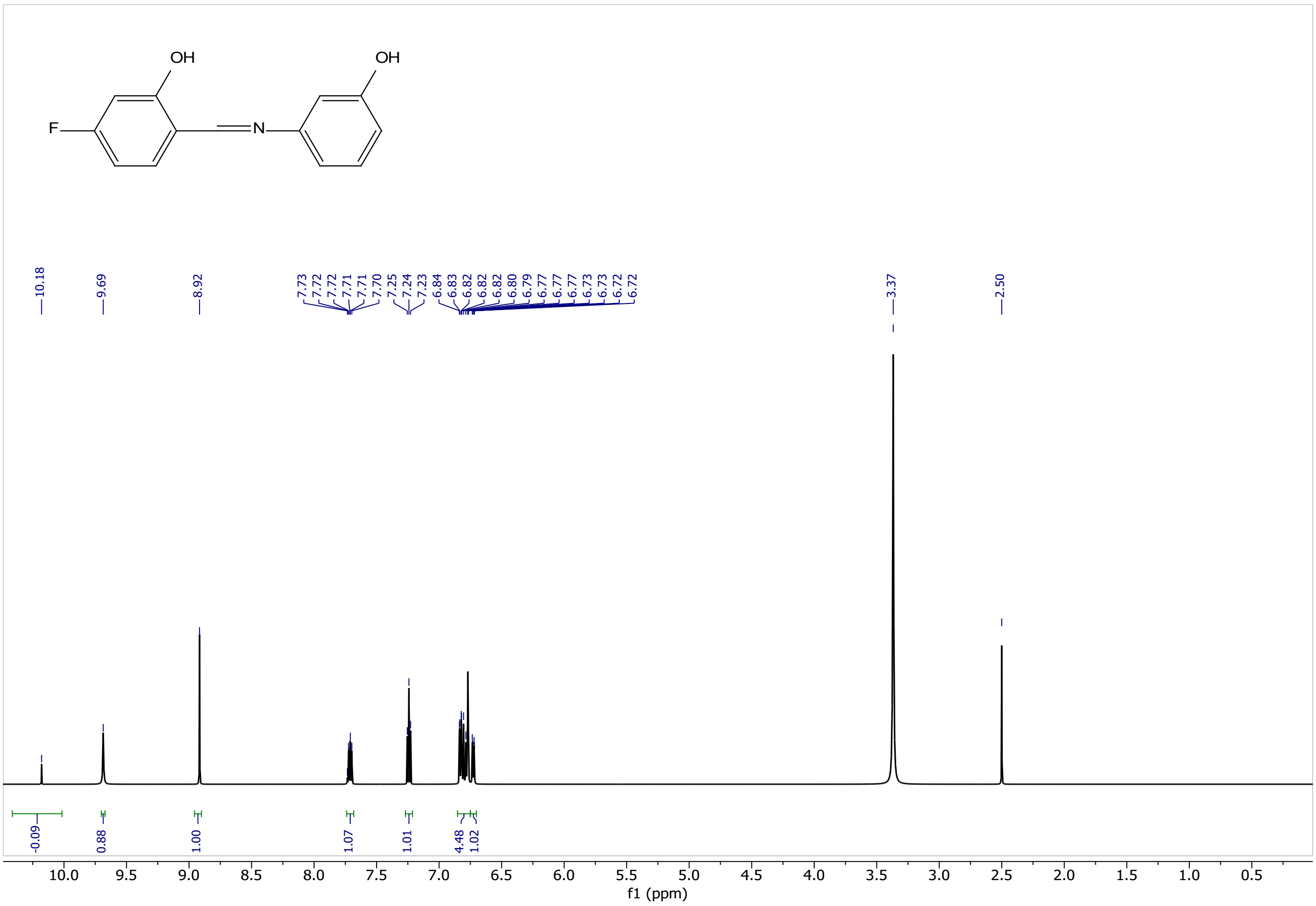

Figure S6. ^{1}H NMR spectrum of M2

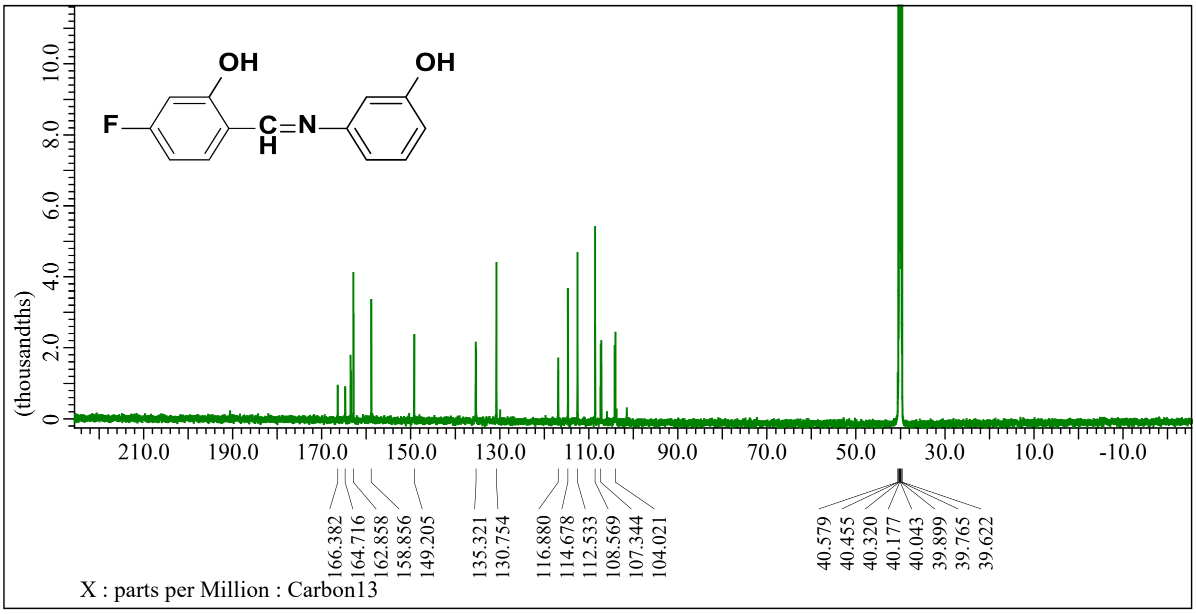

Figure S7. ^{13}C NMR spectrum of M2

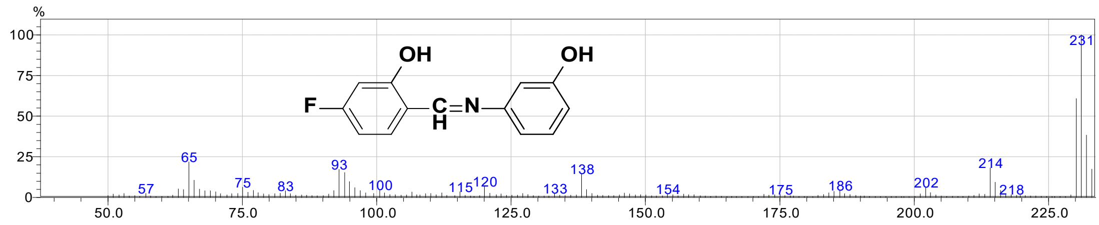

Figure S8. Mass spectrum of M2

3) 2-(((4-Chlorophenyl)imino)methyl)-4-fluorophenol (M3)

Orange; mp: 124-125 °C; Yield: 87%; **IR υ_{max}** (cm^{-1}): 3065 (OH), 1615 (C=N); **^{1}H NMR** (600 MHz, Chloroform-*d*) δ 8.54 (s, C-C**H**=N-, 1H), 7.46 – 7.33 (m, -C**H**-CF-, 2H), 7.29 – 7.18 (m, -C**H**-CCl-, 2H), 7.14 – 7.07 (m, =N-C-CH-, 2H), 6.98 (dd, *J*= 9.0, 4.4 Hz , -COH-C**H**-, 1H); **^{13}C-NMR** (151 MHz, CHLOROFORM-*d*) δ 161.9 (C=N), 157.3, 156.5 (C-F), 154.9, 146.7, 133.0, 129.7, 122.6, 120.7, 118.8, 118.6, 117.3, 117.2 (Ar-C); **MS** m/z (%): 249.05 (100), 250.05 (64.68).

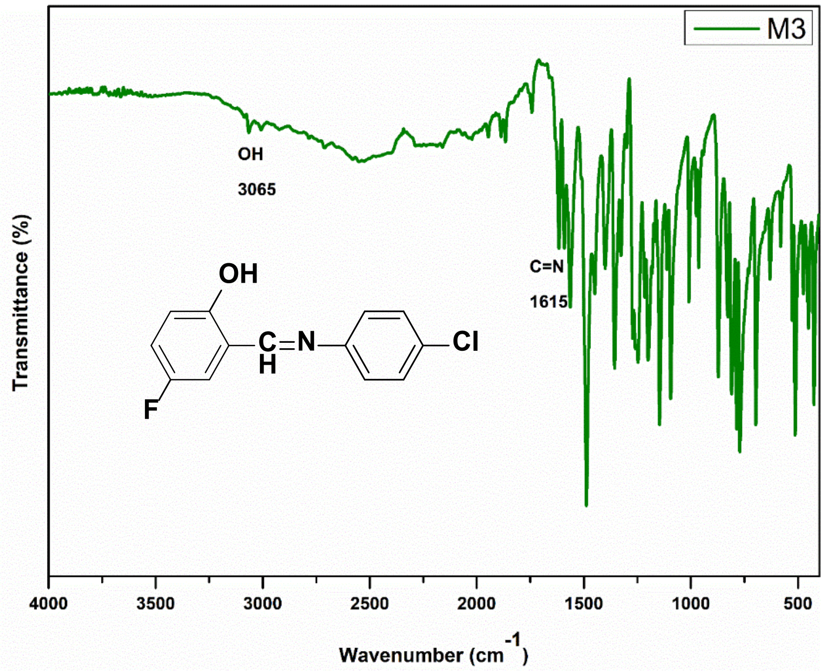

Figure S9. FTIR spectrum of M3

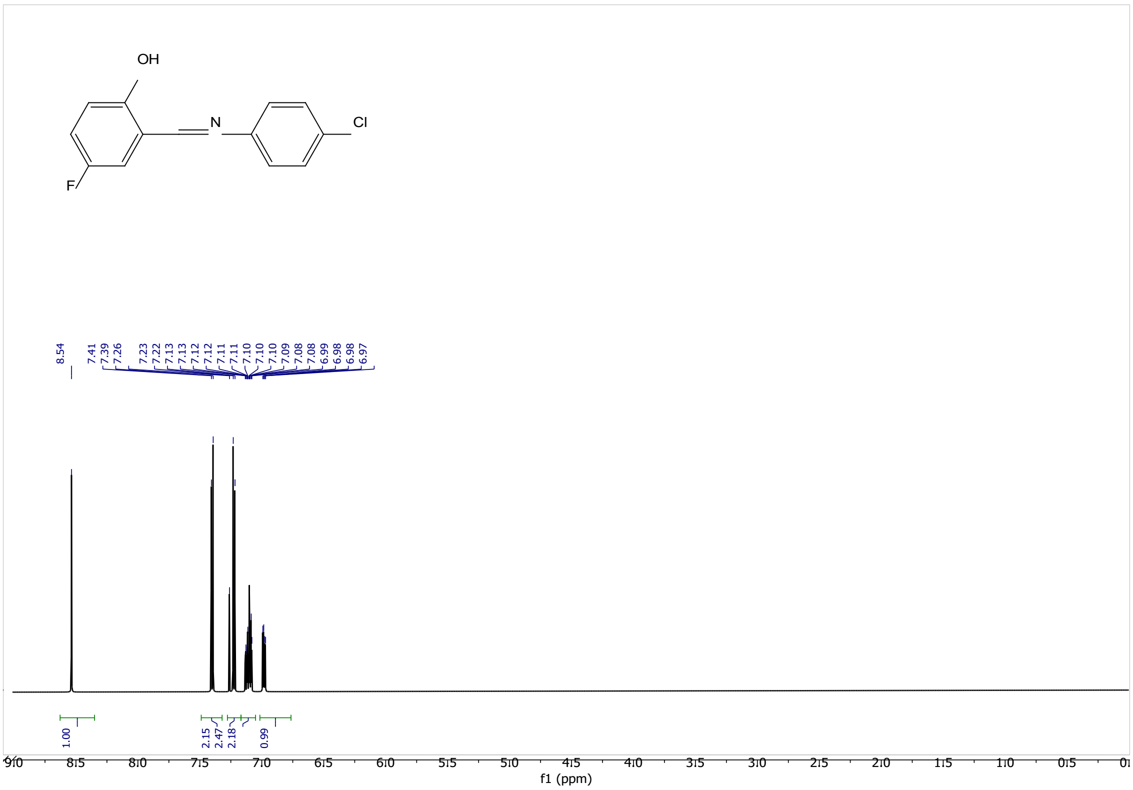

Figure S10. ^{1}H NMR spectrum of M3

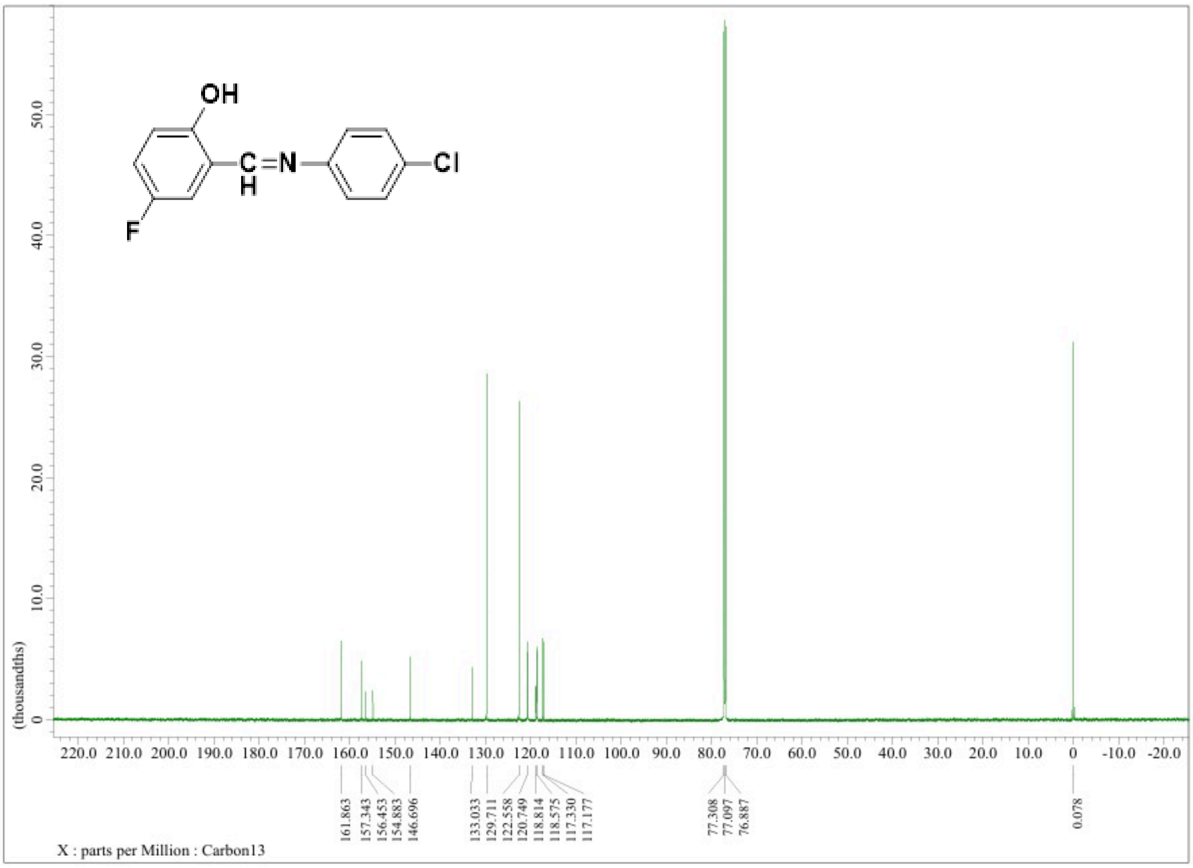

Figure S11. ^{13}C NMR spectrum of M3

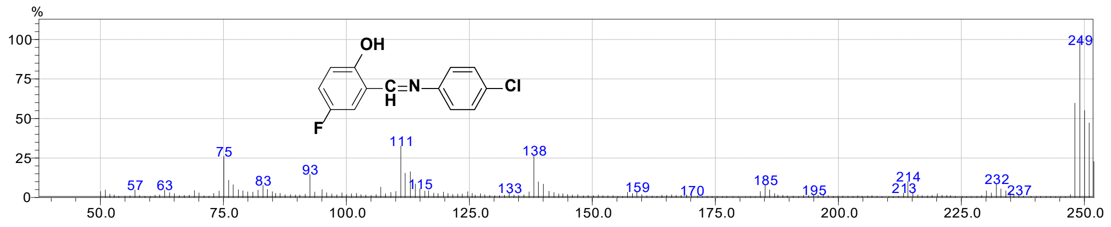

Figure S12. Mass spectrum of M3

4) 2-(((4-Chlorophenyl)imino)methyl)-5-fluorophenol (M4)

Gold; mp: 128-130 °C; Yield: 90%; **IR** υ_{max} (cm^{-1}): 3086 (OH), 1608 (C=N); **^{1}H NMR** (600 MHz, Chloroform-*d*) δ 8.56 (s, -C-C**H**=N-, 1H), 7.41 – 7.37 (m, =CH-C-C**H**-, 3H), 7.21 (d, *J* = 8.7 Hz, =N-C-C**H**-, 3H), 6.71 (dd, *J* = 10.6, 2.5 Hz, -CF-C**H**-CH-, 1H), 6.66 (td, *J* = 8.4, 2.5 Hz, -COH-C**H**-CF-, 1H); **^{13}C-NMR** (151 MHz, CHLOROFORM-*d*) δ 166.9 (C-F), 165.2, 163.5, 162.0 (C=N), 146.7, 134.3, 132.7, 129.7, 122.5, 116.0, 107.3, 107.2, 104.6 (Ar-C); **MS** m/z (%): 249.05 (100), 250.05 (56.93).

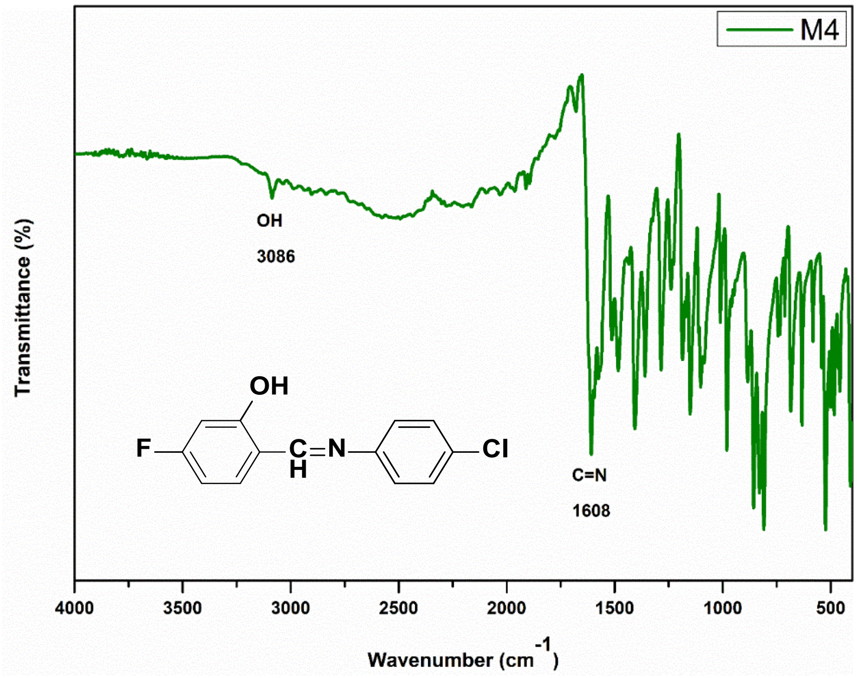

Figure S13. FTIR spectrum of M4

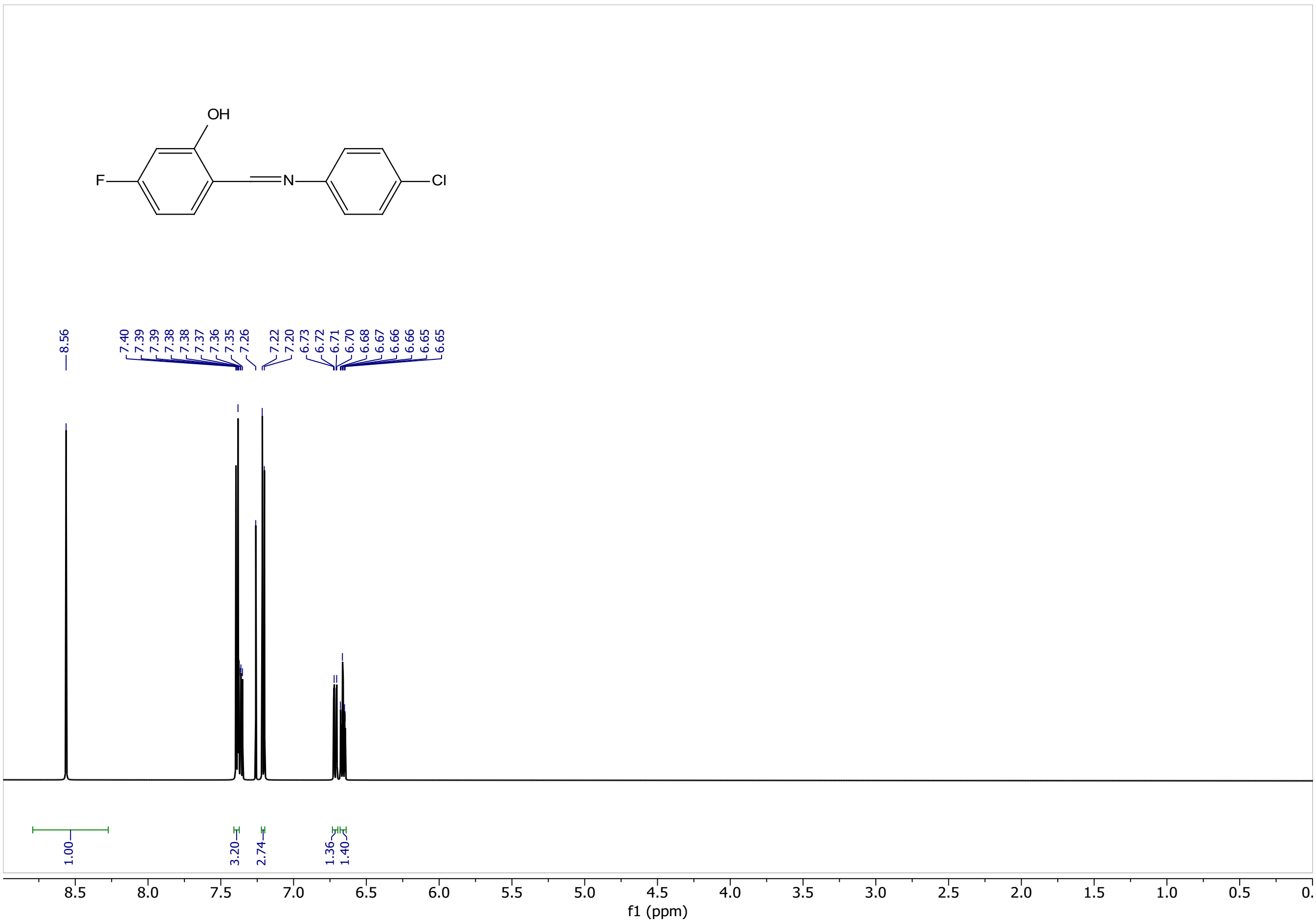

Figure S14. ^{1}H NMR spectrum of M4

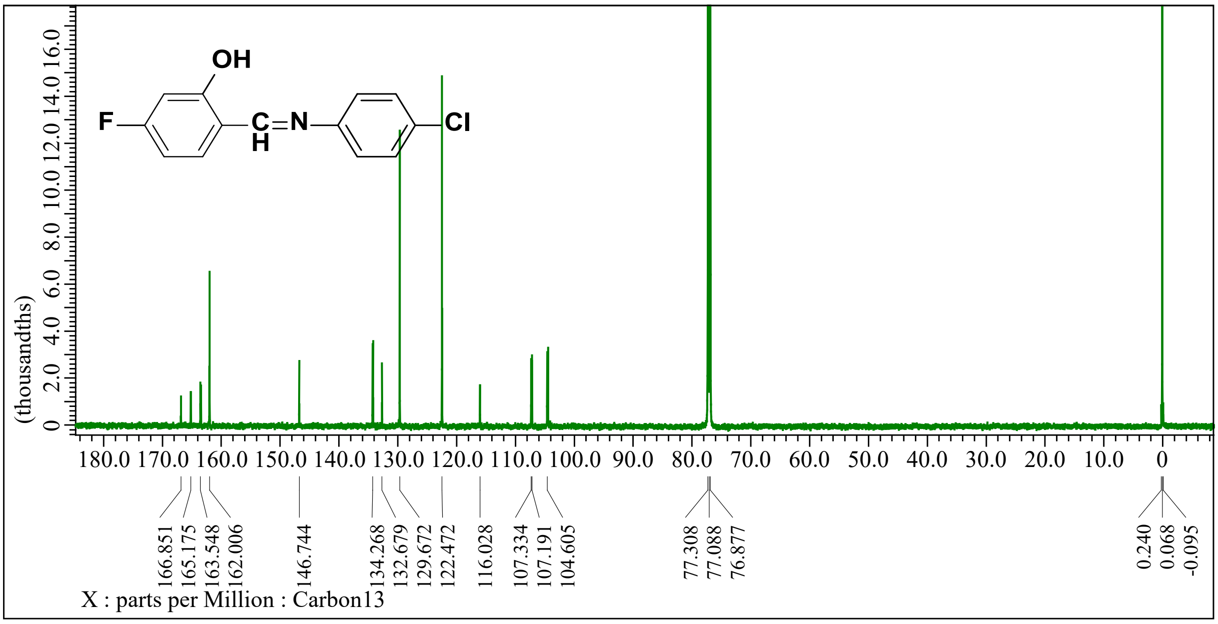

Figure S15. ^{13}C NMR spectrum of M4

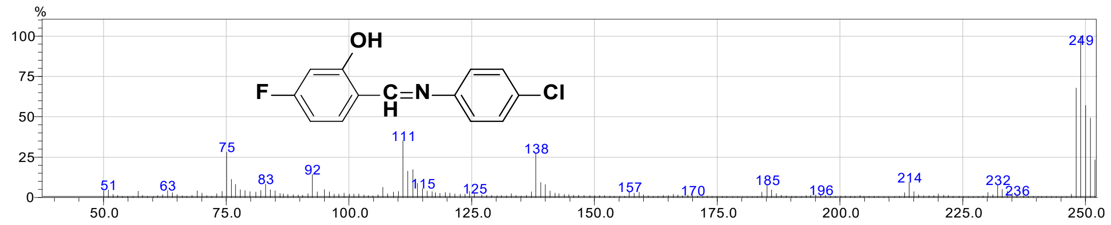

Figure S16. Mass spectrum of M4

5) 1-(3-((5-Fluoro-2-hydroxybenzylidene)amino)phenyl)ethan-1-one (M5)

Yellow; mp: 130-132 °C; Yield: 86%; **IR υ_{max}** (cm^{-1}): 3341 (OH), 1620 (C=N), 1676 (C=O); **^{1}H NMR** (400 MHz, DMSO-d_6) δ 8.99 (s, C-C**H**=N-C-, 1H), 7.95 – 7.84 (m, $COCH_3$-C-C**H**-CH, 1H), 7.66 (d, *J* = 1.8 Hz, -CH-C**H**-CH, 1H), 7.62 (d, *J* = 7.5 Hz, =CH-C-C**H**-CF-, 1H), 7.55 (dd, *J* = 9.0, 3.2 Hz, =N-C-C**H**-C-$COCH_3$, 1H), 7.35 – 7.26 (m, -CF-C**H**-CH-COH-, 1H), 7.18 – 7.07 (m, =N-C-C**H**-CH-, 1H), 7.00 (dd, *J* = 9.1, 4.6 Hz, -COH-C**H**-CH-, 1H), 2.64 (s, -COC**H**$_3$, 3H). **^{13}C-NMR** (151 MHz, DMSO-d_6)δ 198.2 (C=O), 163.2 (C=N), 156.90 (C-F), 149.43, 138.53, 130.39, 127.11, 126.64, 121.29, 118.61, 117.22, 113.18 (Ar-C), 27.41 (CH_3); **MS** m/z (%): 257.15 (100), 258.05 (44.49).

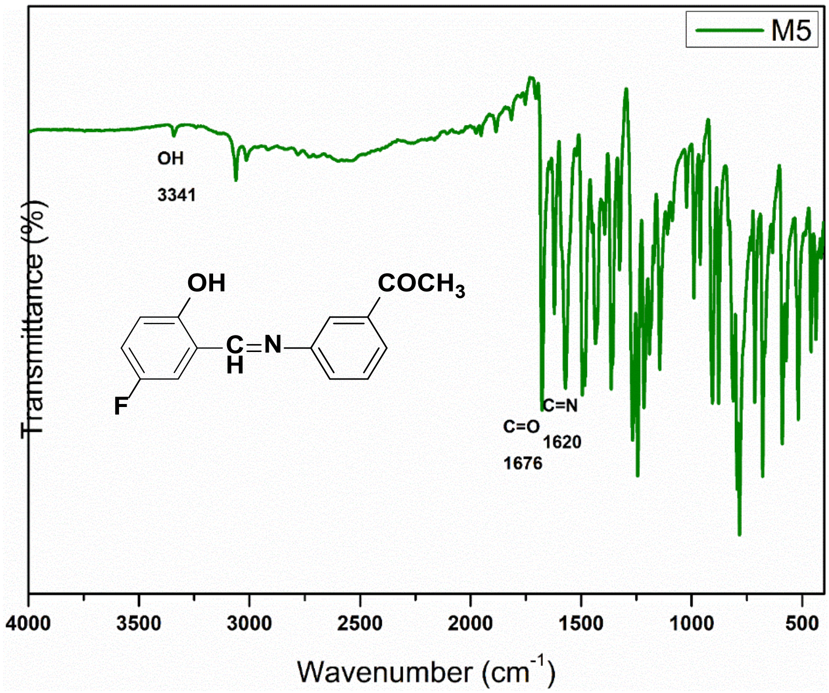

Figure S17. FTIR spectrum of M5

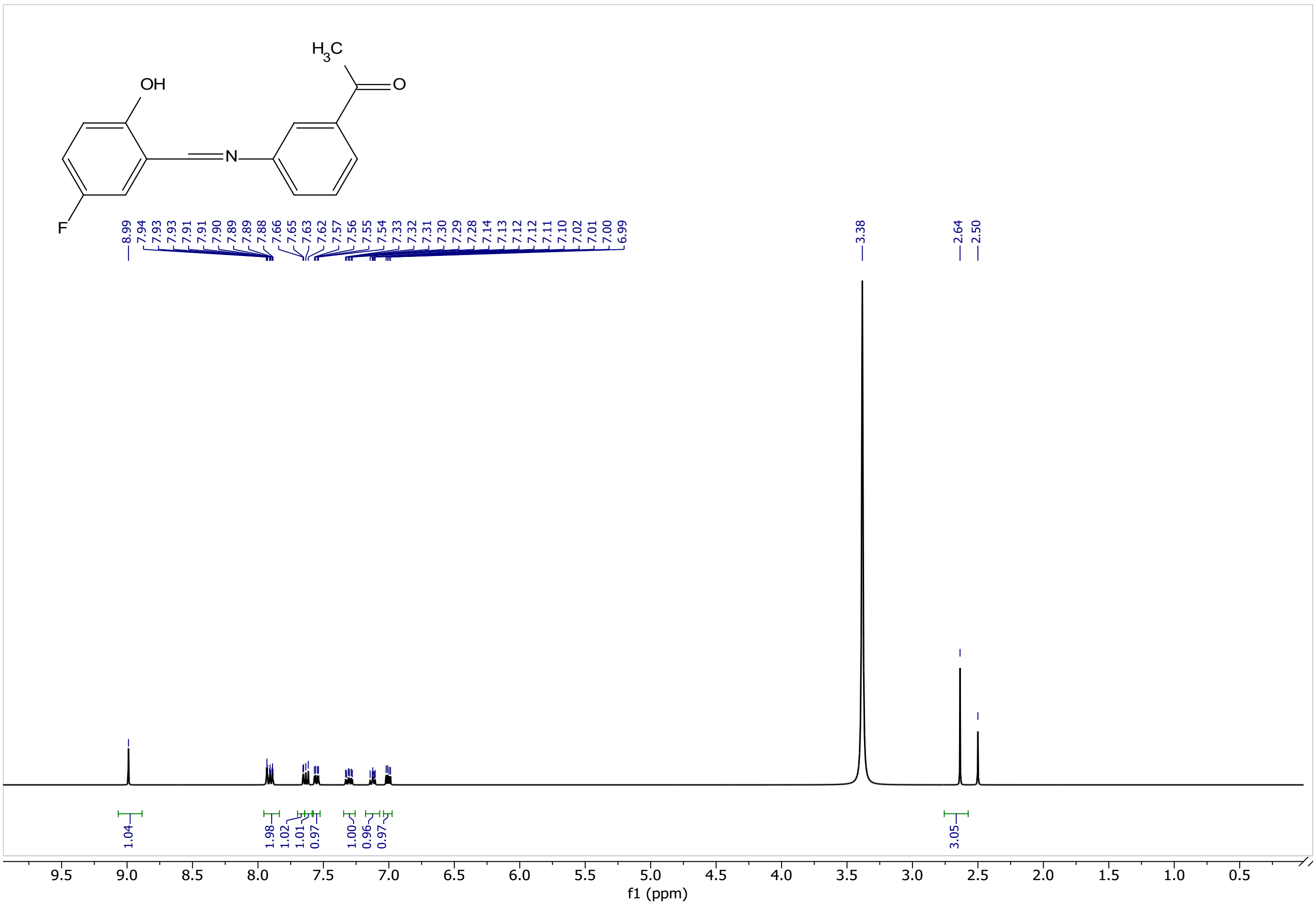

Figure S18. ^{1}H NMR spectrum of M5

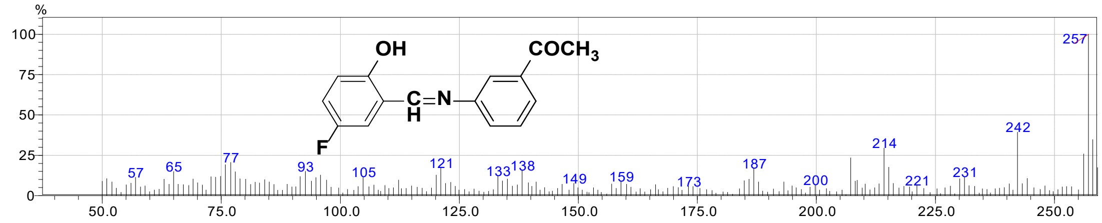

Figure S19. Mass spectrum of M5

6) 1-(3-((4-Fluoro-2-hydroxybenzylidene)amino)phenyl)ethan-1-one (M6)

Dark yellow; mp: 133-135 °C; Yield: 90%; **IR ʋmax** (cm^{-1}): 3081 (OH), 1678 (C=O), 1619 (C=N);**^{1}H NMR** (600 MHz, DMSO-d_6) δ 9.04 (s, -C-C**H**=N-, 1H), 7.95 (t, *J* = 1.9 Hz, =N-C-C**H**-C-COH_3 , 1H), 7.89 (dt, *J* = 7.6, 1.4 Hz, -COCH3-C-C**H**-CH, 1H), 7.76 (dd, *J* = 8.5, 6.8 Hz, 1H), 7.67 (ddd, *J* = 7.9, 2.2, 1.1 Hz, 1H), 7.61 (t, *J* = 7.8 Hz, 1H), 6.90 – 6.79 (m, 1H), 2.64 (s, 3H); **^{13}C-NMR** (151 MHz, DMSO-d_6) δ 198.2 (C=O), 166.5 (C-F), 164.2, 163.2 (C=N), 148.8, 138.6, 135.5, 130.4, 127.0, 126.7, 121.4, 117.0, 107.6, 104.2 (Ar-C), 27.5 (CH_3); **MS** m/z (%): 257.1 (100), 258.05 (42.6).

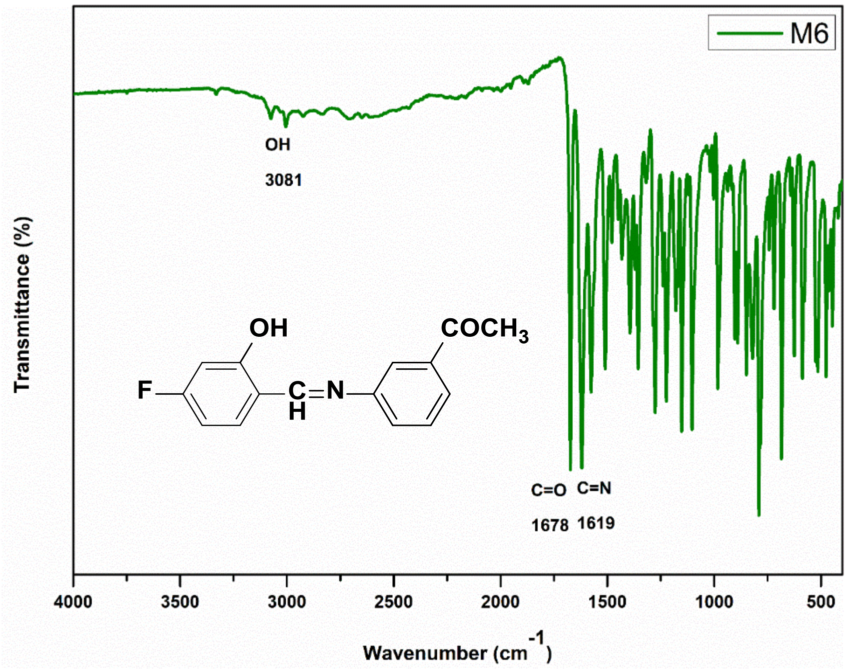

Figure S20. FTIR spectrum of M6

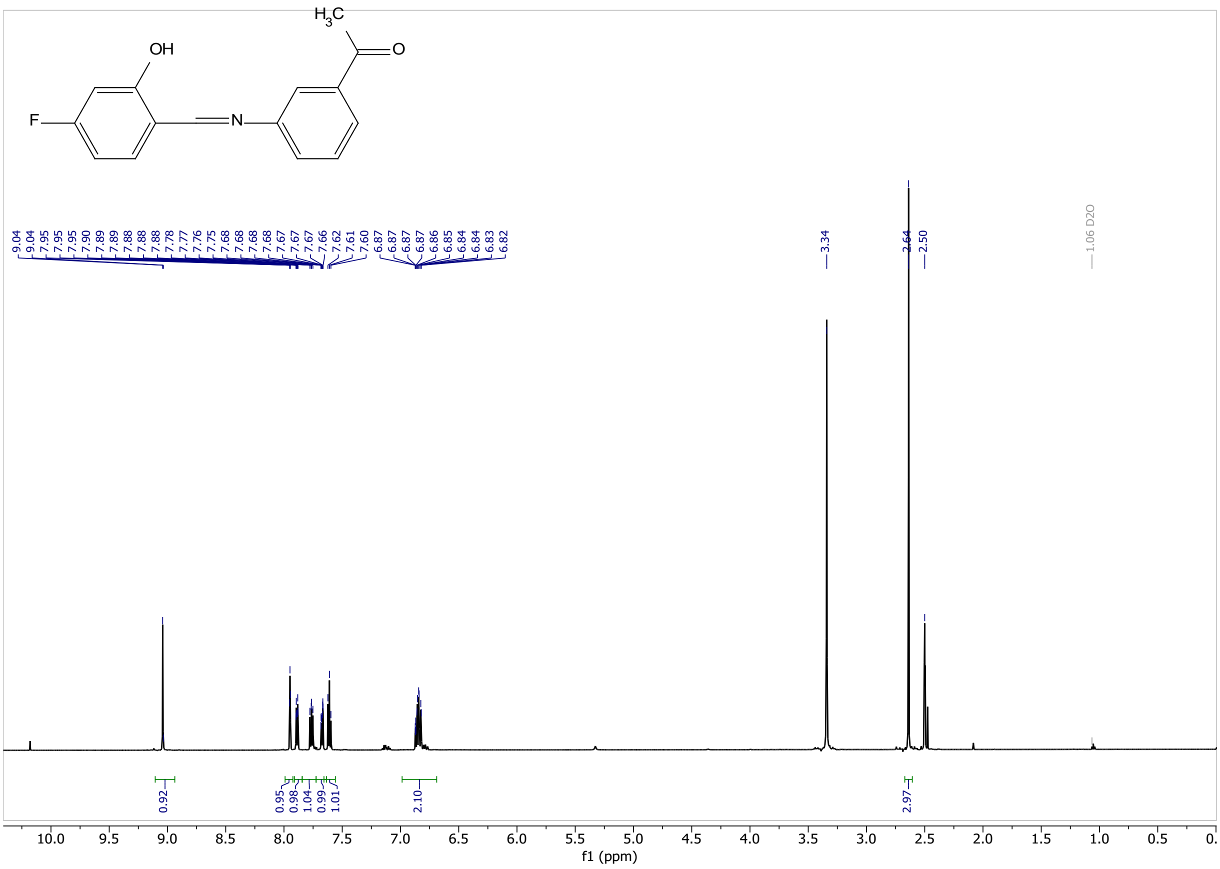

Figure S21. ^{1}H NMR spectrum of M6

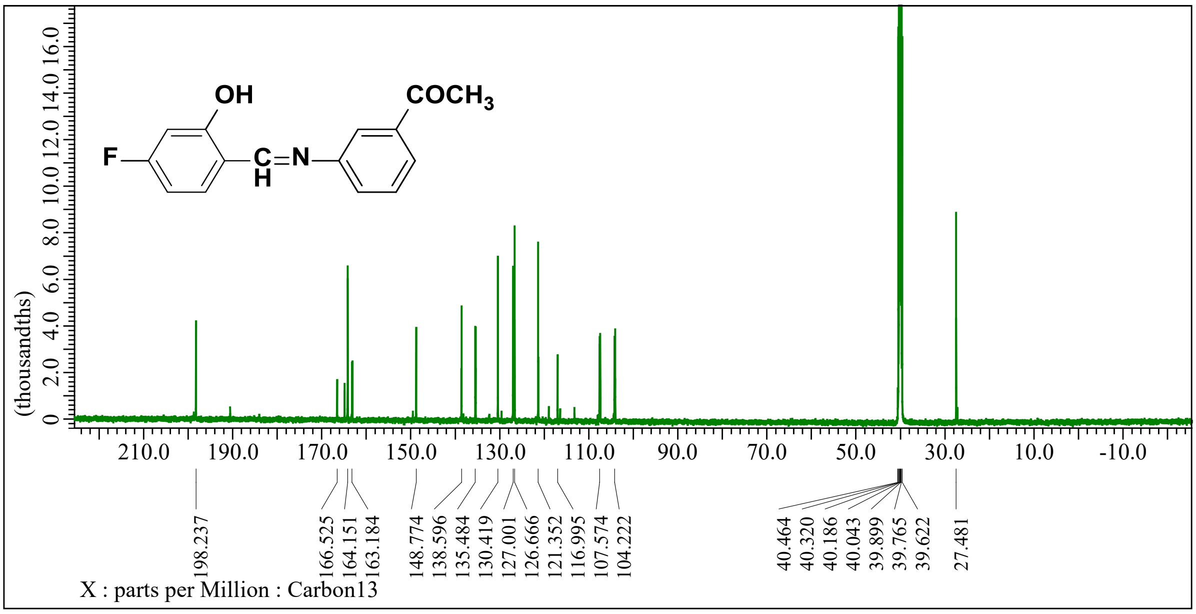

Figure S22. ^{13}C NMR spectrum of M6

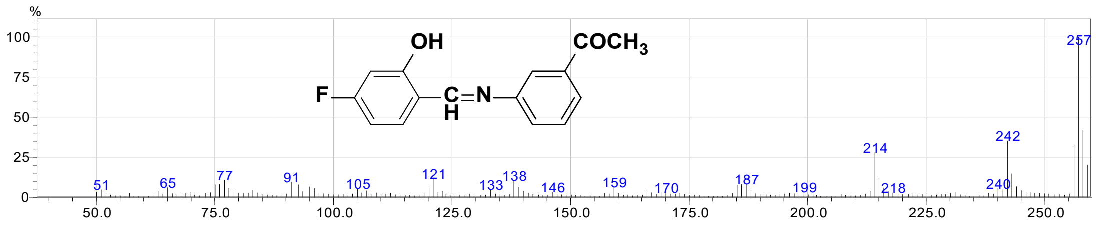

Figure S23. Mass spectrum of M6

7) 4-Fluoro-2-(((2-hydroxyphenyl)imino)methyl)phenol (M7)

Reddish orange; mp: 182-183 °C; Yield: 92%; **IR ʋmax** (cm^{-1}): 3051 (OH), 1628 (C=N); ^{1}**H NMR** (600 MHz, DMSO-*d*$_6$) δ 9.77 (s, =N-C-CO**H**-, 1H), 8.95 (s, -C-C**H**=N-, 1H), 7.51 (dt, *J* = 9.0, 2.4 Hz, -C-C**H**-CF-, 1H), 7.34 (dt, *J* = 7.9, 1.3 Hz, -CF-C**H**-CH-, 1H), 7.30 – 7.18 (m, -CH-C**H**-CH-CH-, 1H), 7.19 – 7.10 (m, -CH-CH-CH-C**H**-, 1H), 7.14 (d, *J* = 1.6 Hz, 1H), 7.06 – 6.92 (m, =N-C-CH-C**H**-CH-, 2H), 6.91 – 6.83 (m, -COH-C**H**-, 1H); ^{13}**C-NMR** (151 MHz, DMSO-*d*$_6$) δ 160.7 (C=N), 157.4, 156.1 (C-F), 154.5, 151.8, 135.3, 128.9, 120.1, 120.0, 118.5, 117.5, 117.3, 117.1 (Ar-C)**; MS** m/z (%): 231.1 (96.94), 232.05 (35.21).

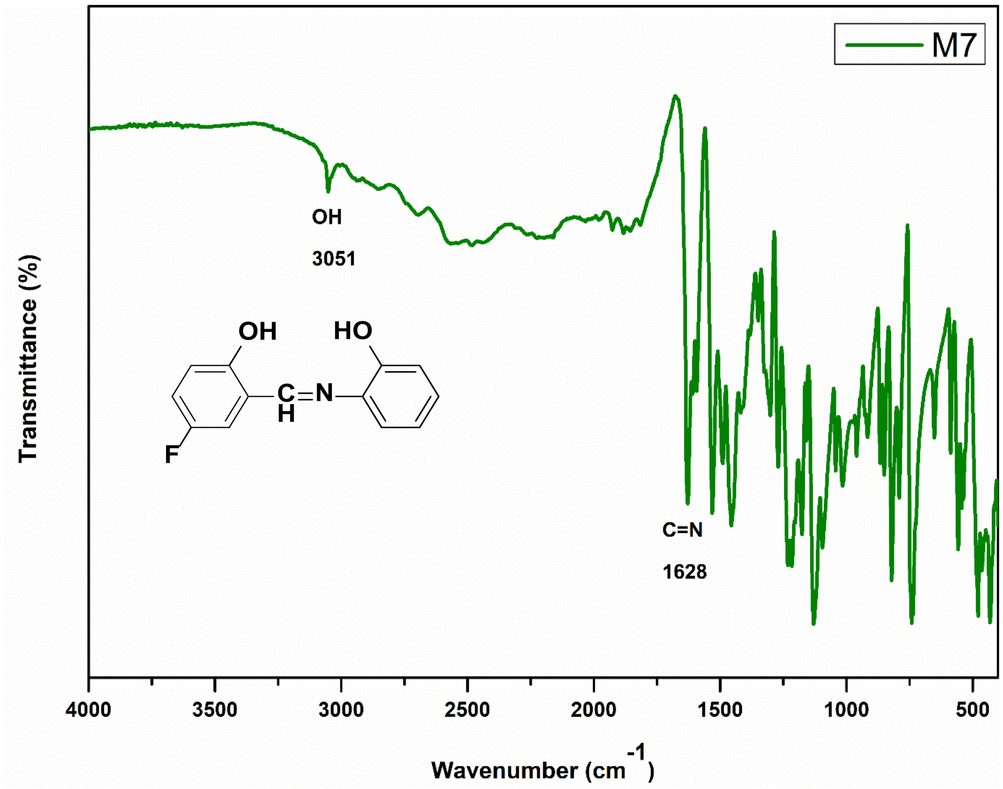

Figure S24. FTIR spectrum of M7

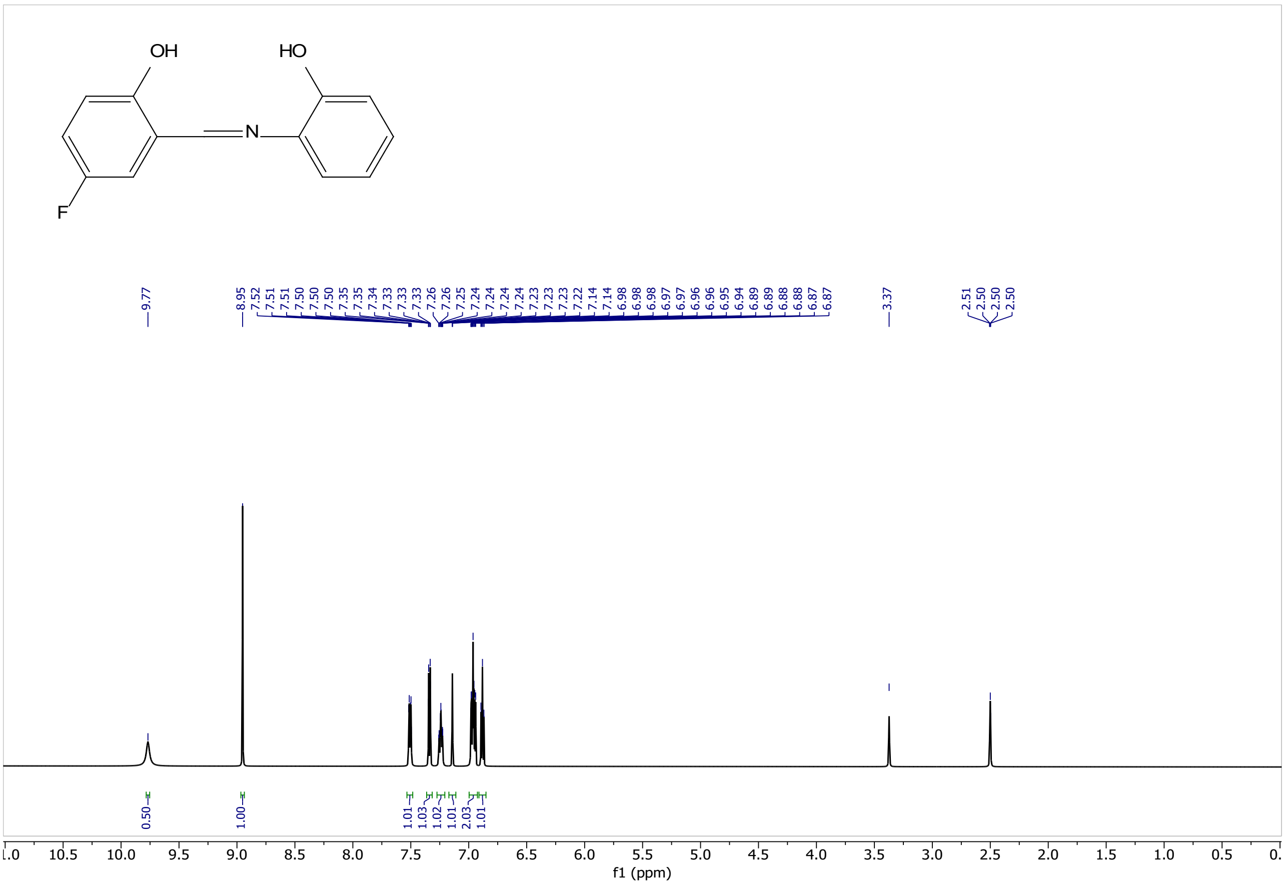

Figure S25. ^{1}H NMR spectrum of M7

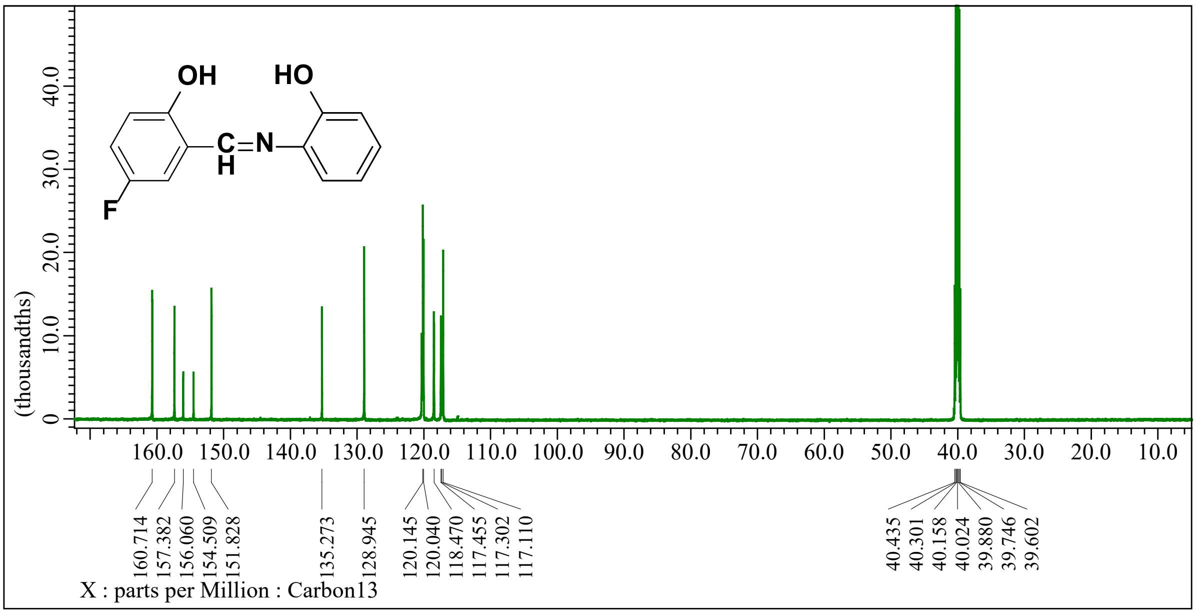

Figure S26. ^{13}C NMR spectrum of M7

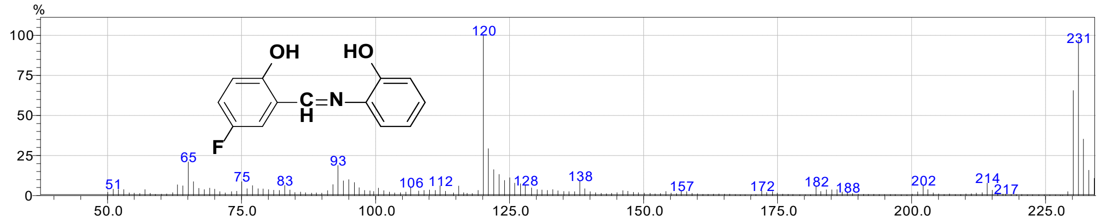

Figure S27. Mass spectrum of M7

8) 5-Fluoro-2-(((2-hydroxyphenyl)imino)methyl)phenol (M8)

Gold; mp: 187-189 °C; Yield: 87%; **IR ʋmax** (cm^{-1}): 3062 (OH), 1614 (C=N); **^{1}H NMR** (600 MHz, DMSO-d_6) δ 9.86 (s, =N-C-CO**H** -,1H), 8.99 (s, -C-C**H**=N-, 1H), 7.65 (dd, J = 8.6, 7.0 Hz, =CH-C-C**H**-, 1H), 7.39 (dd, J = 8.0, 1.6 Hz, -COH-CH-C**H**-, 1H), 7.13 (ddd, J = 8.1, 7.3, 1.6 Hz, =N-C-C**H**-, 1H), 6.97 (dd, J = 8.1, 1.4 Hz, -COH-C**H**-, 1H), 6.88 (td, J = 7.6, 1.4 Hz, -COH-C**H**-CF-, 1H), 6.83 – 6.58 (m, =N-C-CH-C**H**-CH-, 2H); **^{13}C-NMR** (151 MHz, DMSO-d_6) δ 166.5 (C-F), 165.3, 164.9, 160.8 (C=N), 151.4, 135.2, 134.2, 128.7, 120.2, 119.8, 117.0, 106.6, 104.3 (Ar-C); **MS** m/z (%): 231.1 (100), 232.05 (37.06).

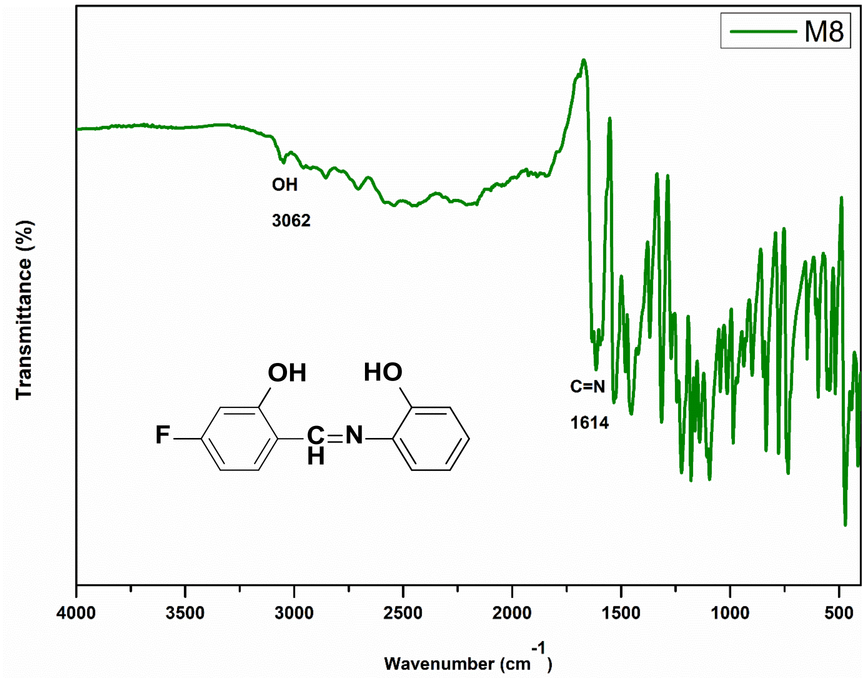

Figure S28. FTIR spectrum of M8

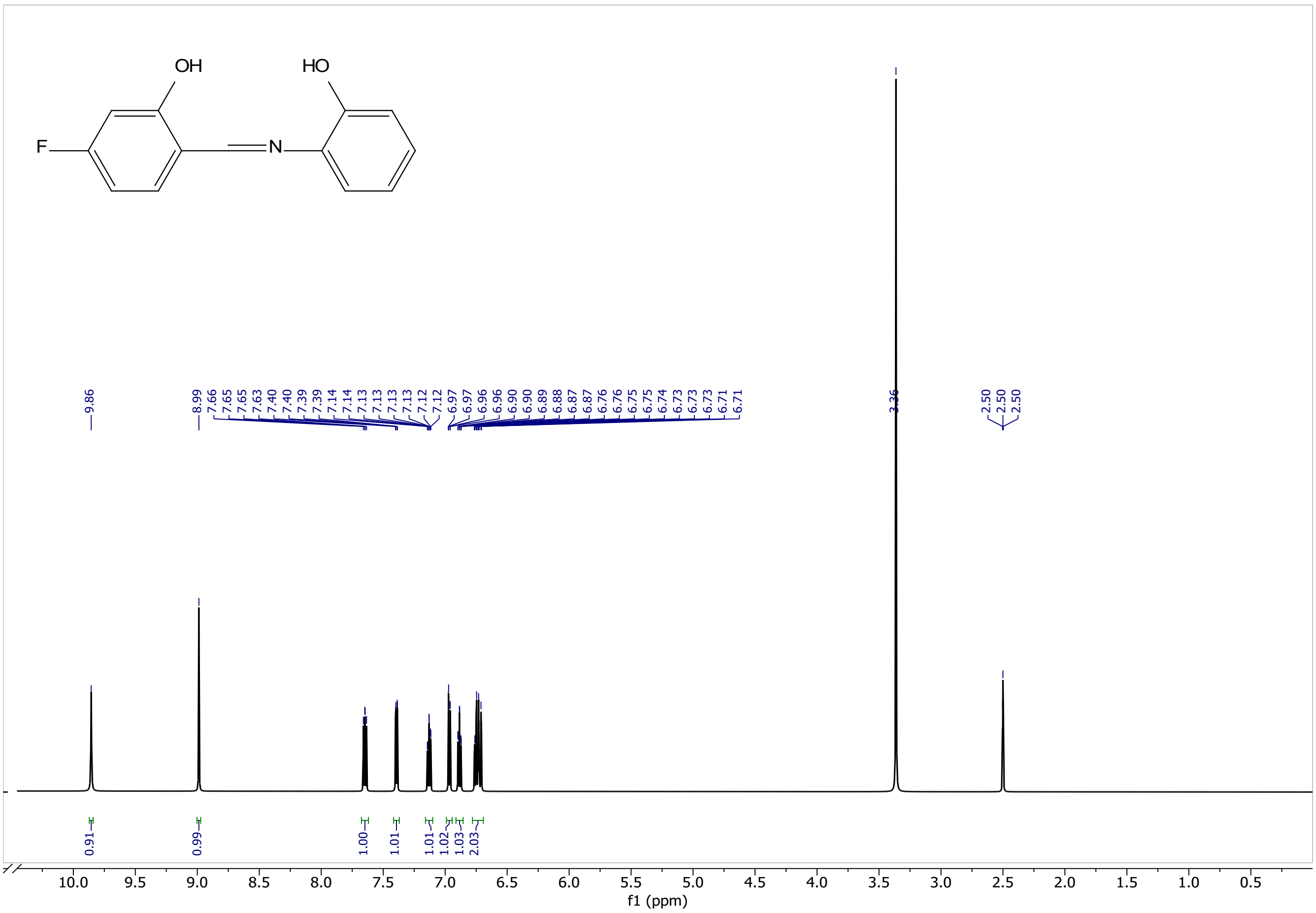

Figure S29. ^{1}H NMR spectrum of M8

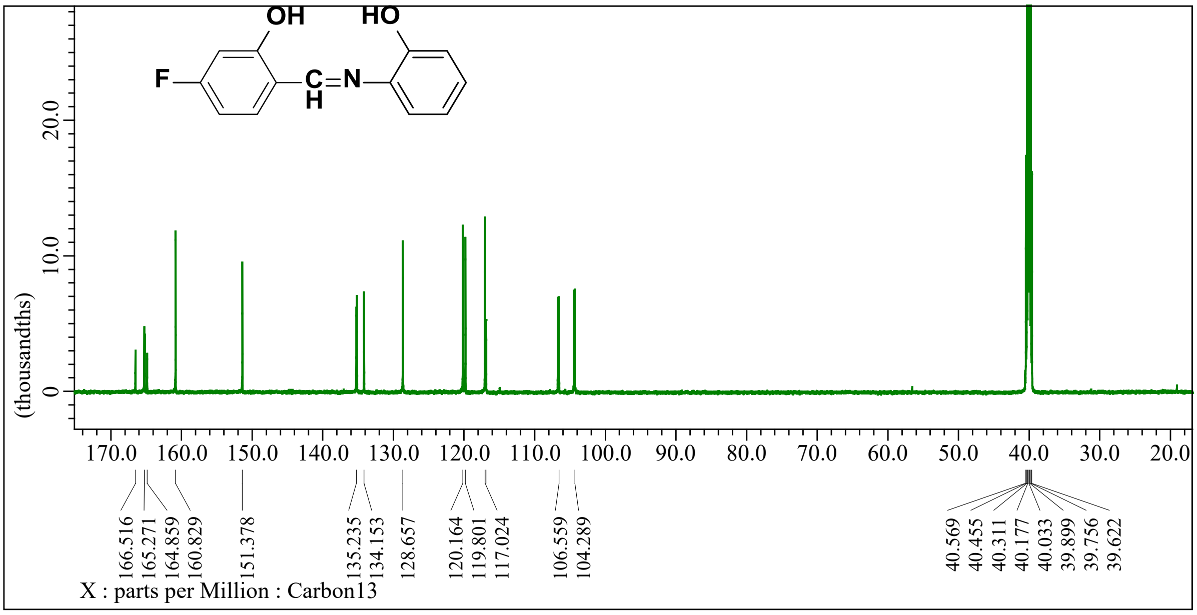

Figure S30. ^{13}C NMR spectrum of M8

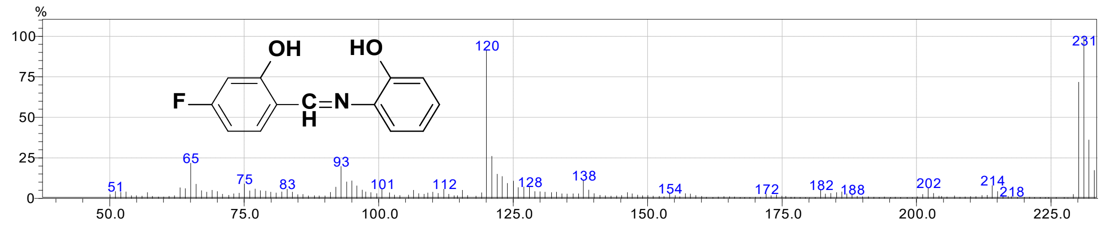

Figure S31. Mass spectrum of M8

9) 2,2'-((1*E*,1'*E*)-(1,2-phenylenebis(azaneylylidene))bis(methaneylylidene))bis(4-fluorophenol) (M9)

Yellow; mp: 129-131 °C; Yield: 88%; **IR υ_{max}** (cm^{-1}): 3357 (OH), 1614 (C=N); **^{1}H NMR** (600 MHz, Chloroform-*d*) δ 8.57 (s, -C-N=C**H**-C, 2H), 7.4-7.34 (m, -CF-C**H**-C-CH=, 2H), 7.28 – 7.22 (m, N-C-CH-C**H**- , 2H), 7.13 – 7.06 (m, =N-C-C**H**-, 4H), 7.01 (dd, *J* = 8.9, 4.5 Hz, =CH-C-COH-C**H**-, 2H); **^{13}C-NMR** (151 MHz, CHLOROFORM-*d*) δ 162.7 (2C=N), 157.6, 156.4 (2C-F), 154.8, 142.3, 128.2, 120.8, 120.6, 119.7, 118.9, 118.88, 118.83, 118.78, 117.2, 117.1 (Ar-C); MS m/z (%): 352(13), 350 (50).

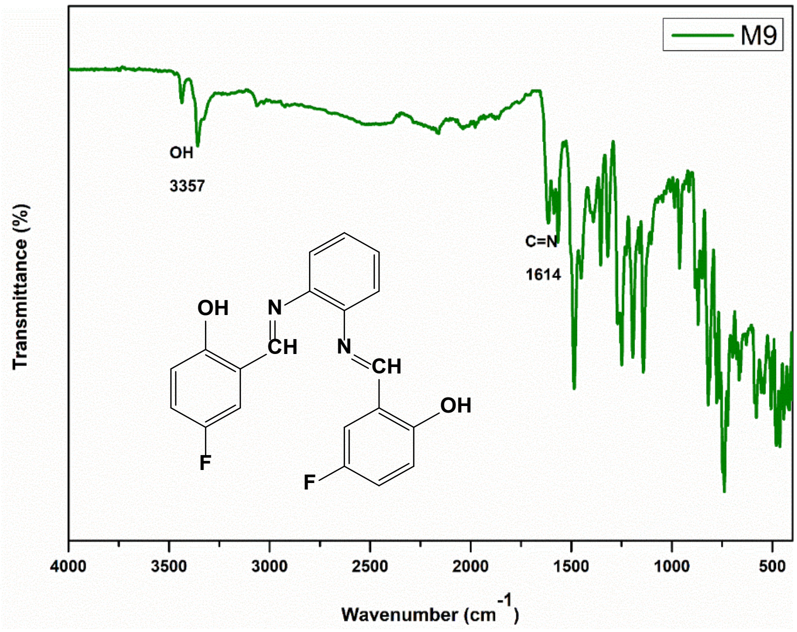

Figure S32. FTIR spectrum of M9

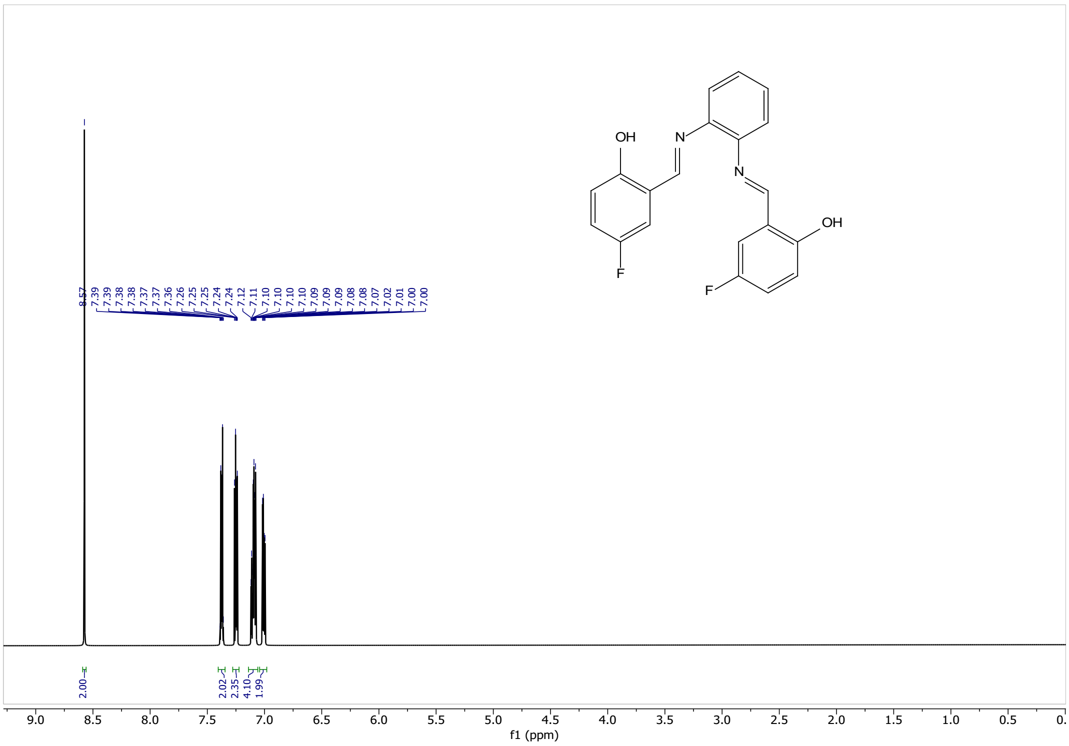

Figure S33. ^{1}H NMR spectrum of M9

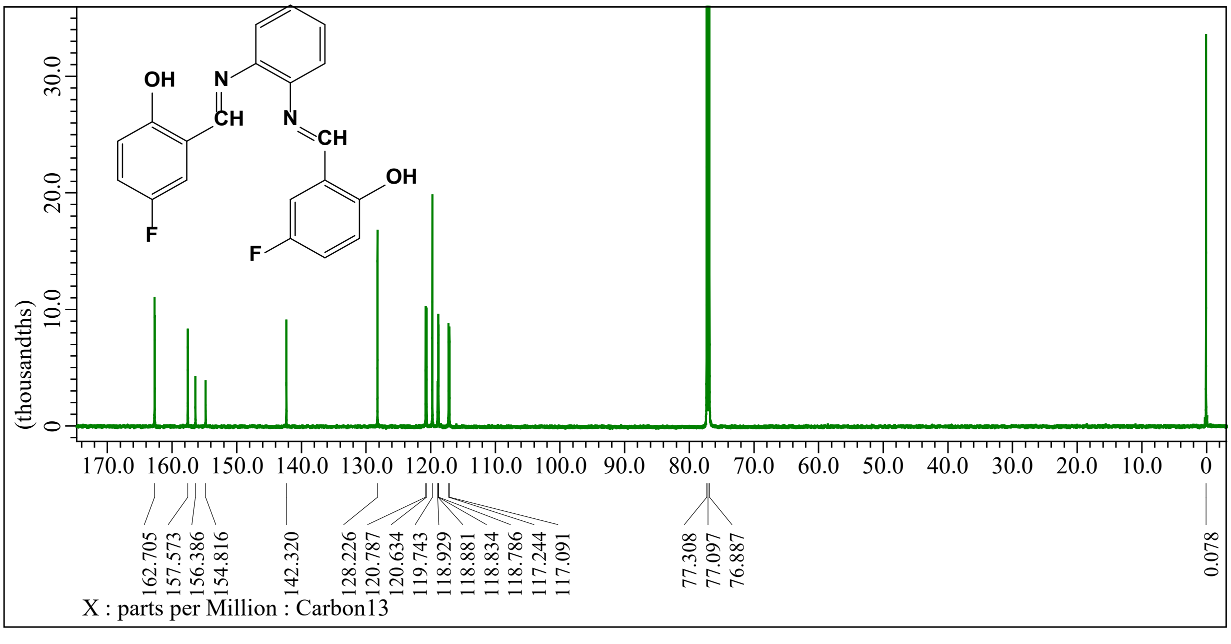

Figure S34. ^{13}C NMR spectrum of M9

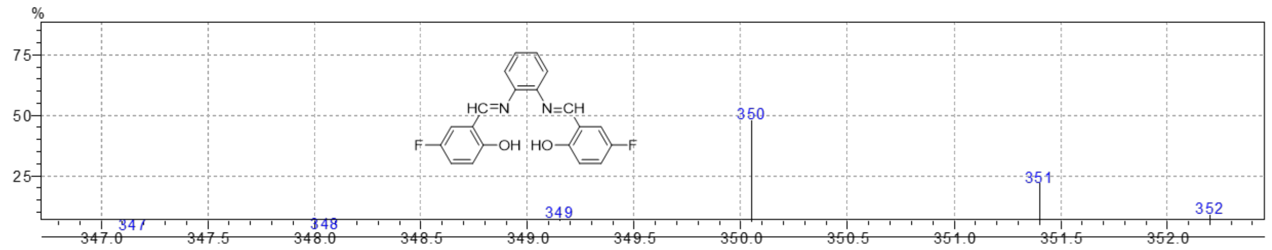

Figure S35. Mass spectrum of M9

10) 6,6'-((1*E*,1'*E*)-(1,2-phenylenebis(azaneylylidene))bis(methaneylylidene))bis(3-fluorophenol) (M10)

Orange; mp: 131-133 °C; Yield: 89%; **IR** υ_{max} (cm^{-1}): 3387 (OH), 1608 (C=N); **^{1}H NMR** (600 MHz, DMSO-d_6) δ 8.95 (s,-C-C**H**=N-, 2H), 7.84 – 7.67 (m, =CH-C-C**H**-, 2H), 7.48 (dd, *J* = 6.0, 3.4 Hz, =N-C-CH-C**H**-, 2H), 7.40 (dd, *J* = 5.9, 3.4 Hz, =N-C-C**H**-, 2H), 6.91 – 6.71 (m, -CF-C**H**-, 4H); **^{13}C-NMR** (151 MHz, DMSO-d_6) δ 166.6 (2C-F), 164.9, 163.6, 163.5 (2C=N), 142.2, 135.24, 135.16, 128.4, 120.2, 117.2, 107.4, 107.2, 104.3, 104.1 (Ar-C); **MS** m/z (%): 352.1 (42.07), 353.05 (19.51).

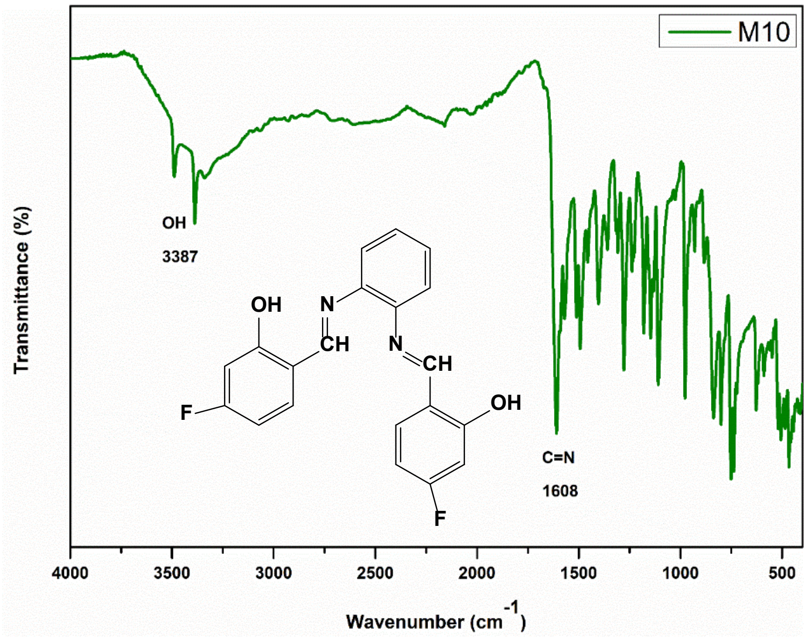

Figure S36. FTIR spectrum of M10

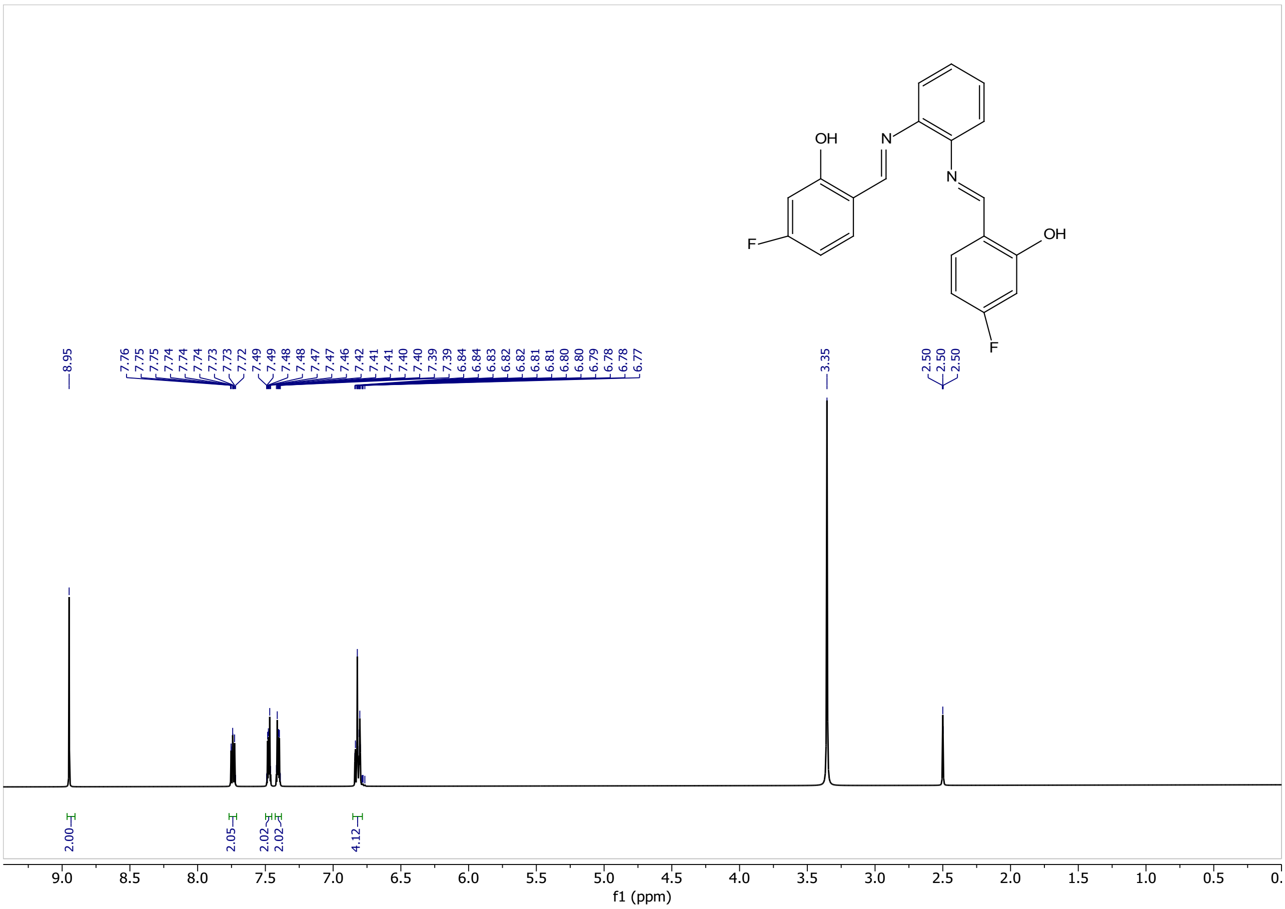

Figure S37. ^{1}H NMR spectrum of M10

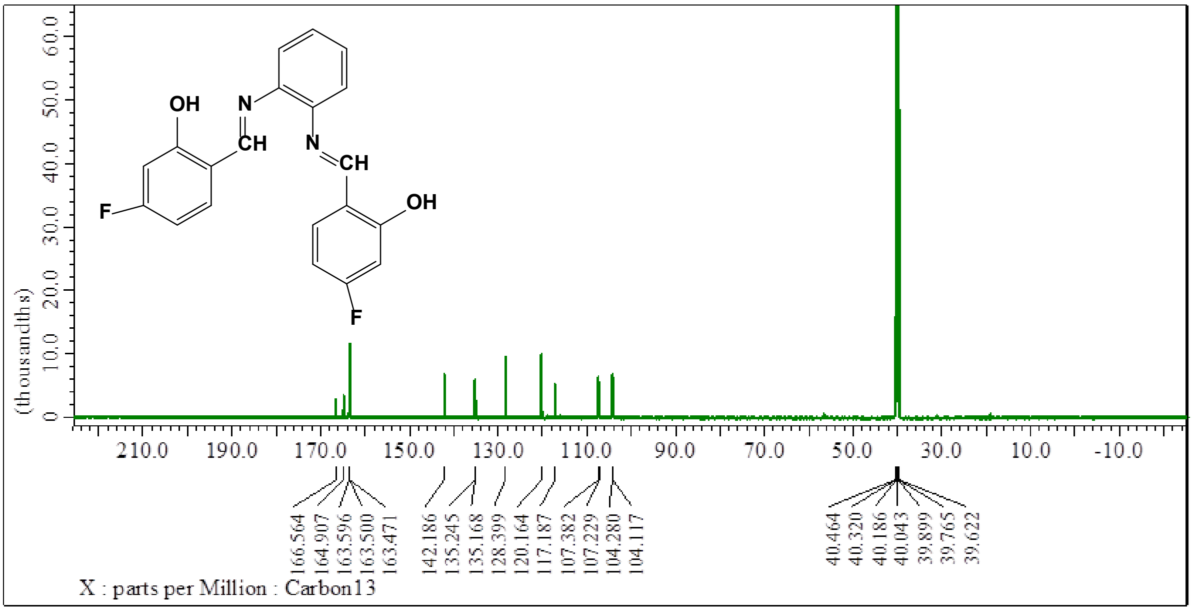

Figure S38. ^{13}C NMR spectrum of M10

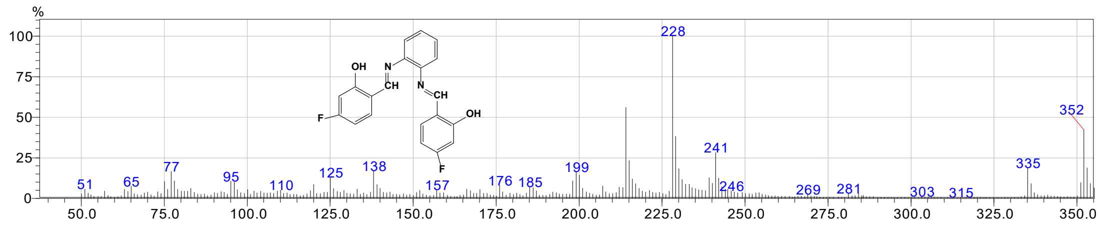

Figure S39. Mass spectrum of M10
